# Zinc is a potent and specific inhibitor of IFN-λ3 signalling

**DOI:** 10.1038/ncomms15245

**Published:** 2017-05-17

**Authors:** Scott A. Read, Kate S. O'Connor, Vijay Suppiah, Chantelle L. E. Ahlenstiel, Stephanie Obeid, Kristina M. Cook, Anthony Cunningham, Mark W. Douglas, Philip J. Hogg, David Booth, Jacob George, Golo Ahlenstiel

**Affiliations:** 1 Storr Liver Centre, The Westmead Institute for Medical Research, The University of Sydney and Westmead Hospital, Westmead, New South Wales 2145, Australia; 2 Centre for Immunology and Allergy Research, The Westmead Institute for Medical Research, The University of Sydney and Westmead Hospital, Westmead, New South Wales 2145, Australia; 3 School of Pharmacy and Medical Science, University of South Australia, Adelaide, South Australia 5001, Australia; 4 The Kirby Institute for Infection and Immunity in Society, University of New South Wales, Sydney, New South Wales 2052, Australia; 5 The Centenary Institute, Camperdown, New South Wales 2050, Australia; 6 Centre of Virus Research, Westmead Institute for Medical Research, The University of Sydney and Westmead Hospital, Westmead, New South Wales 2145, Australia; 7 Centre for Infectious Diseases and Microbiology, Marie Bashir Institute for Infectious Diseases and Biosecurity, University of Sydney at Westmead Hospital, Westmead, New South Wales 2145, Australia; 8 National Health and Medical Research Council Clinical Trials Centre, University of Sydney, Sydney, New South Wales 2006, Australia

## Abstract

Lambda interferons (*IFNL*, IFN-λ) are pro-inflammatory cytokines important in acute and chronic viral infection. Single-nucleotide polymorphisms *rs12979860* and *rs8099917* within the *IFNL* gene locus predict hepatitis C virus (HCV) clearance, as well as inflammation and fibrosis progression in viral and non-viral liver disease. The underlying mechanism, however, is not defined. Here we show that the *rs12979860* CC genotype correlates with increased hepatic metallothionein expression through increased systemic zinc levels. Zinc interferes with IFN-λ3 binding to IFNL receptor 1 (IFNLR1), resulting in decreased antiviral activity and increased viral replication (HCV, influenza) *in vitro*. HCV patients with high zinc levels have low hepatocyte antiviral and inflammatory gene expression and high viral loads, confirming the inhibitory role of zinc *in vivo*. We provide the first evidence that zinc can act as a potent and specific inhibitor of IFN-λ3 signalling and highlight its potential as a target of therapeutic intervention for IFN-λ3-mediated chronic disease.

## Abstract (web summary)

Lambda interferons (IFNL) are involved in the immune response to viral infection. Here the authors show that zinc can interfere with IFNL signalling, and that in HCV patients the rs12979860 polymorphism regulates blood zinc levels and, subsequently, the hepatic immune response.

The past decade of research has demonstrated that different tissues evoke distinct and tightly regulated antiviral responses based on multiple environmental and host factors: proximity and concentration of antigen, cytokine responsiveness mediated by receptor expression, resident immune cells and genetic background, to name a few. Interferon lambdas (IFN-λs, type III interferons), comprising IFN-λ1, 2, 3 and 4 have only recently been recognized as antiviral and pro-inflammatory cytokines. Although IFN-λs are beneficial in response to acute infection^1^,^2^,^3^,^4^,^5^,^6^,^7^,^8^, evidence indicates that IFN-λs may drive chronic inflammation and end-organ damage such as hepatic cirrhosis^9^,^10^,^11^,^12^,^13^.

Due to the restricted expression of the IFN-λ receptor (IFNLR1), tissues high in epithelial cell content that are consistently challenged by pathogens, including the lungs, gut and liver, are selectively responsive to IFN-λs^14^. In blood, primary pathogen responders, macrophages and dendritic cells, are responsive to IFN-λs. The receptor complex for type III interferons is unique, consisting of the IL-10 receptor β subunit (IL-10Rβ) and IFN-λR1; however, downstream signalling is similar to type I interferons. Upon activation, the receptor mediates phosphorylation of STAT1 and STAT2, which heterodimerize, and with the addition of IRF9, form the interferon stimulated gene factor 3 (ISGF3). Unlike type I interferons, IFN-λs may require unique accessory signalling pathways (for example, MAPKs) and intermediates (for example, JAK2) for optimal signal transduction, which may explain the prolonged ISG expression mediated by IFN-λs, compared to IFN-α (refs 15, 16, 17).

The importance of IFN-λs became evident after simultaneous genome-wide association studies that identified a cluster of single-nucleotide polymorphisms (SNPs) in the *IFN-λ* gene locus predicting hepatitis C virus (HCV) clearance spontaneously or in response to IFN-α and ribavirin treatment in patients with genotype 1 HCV infection^1^,^2^,^3^,^4^. The *IFNL* genomic locus has since been studied in greater detail, revealing a novel SNP (*rs368234815*) in strong linkage disequilibrium with *rs12979860* that generates IFN-λ4 (ref. 18). IFN-λ4 expression has only been shown in hepatocytes and in individuals possessing the frame shift mutation ΔG at *rs368234815,* instead of the TT allele, which results in an early stop codon. Individuals possessing the favourable *IFN-λ* ‘responder' genotype (*rs12979860* CC, *rs368234815* TT and *rs8099917* TT) maintain lower basal ISG expression in the liver, possibly due to lack of a functional IFN-λ4, and as a result are more responsive to IFN-α treatment^19^,^20^. Another SNP within the IFN-λ4 protein coding region that converts a proline to a serine residue (*rs117648444*) was found to further improve rapid virological response^21^. The IFN-λ4 carrying the serine residue showed decreased ISG induction potential, favouring reduced hepatic ISG pre-activation and subsequently improved HCV clearance.

A functional understanding of how the *IFN-λ* polymorphisms affect the antiviral response and inflammation has not yet been conclusively determined. Differences in IFN-λ3 expression have however been noted by several groups^2^,^3^,^22^. IFN-λ3 affects not only hepatic tissue, as described for IFN-λ4, but also infiltrating immune cells, important mediators of HCV clearance and chronic inflammatory disease in the liver^23^. We have recently shown the *IFNL rs12979860* SNP is a strong predictor of hepatic inflammation and thus progressive end-organ damage in viral (that is, hepatitis B virus (HBV) and HCV infection) and non-viral chronic liver diseases (non-alcoholic steatohepatitis)^23^. Thus, although protective in acute infection, IFN-λs may promote ongoing inflammation and end-organ damage in chronic disease. This type of immune dysfunction has been well characterized in HIV patients, where chronic IFN-α production contributes to numerous HIV-related co-morbidities^24^.

We previously demonstrated that multiple members of the *MT1* metallothionein gene family are highly expressed in the liver of individuals possessing the favourable *IFNL rs8099917* TT responder genotype, the opposite trend to antiviral ISGs, which are low^20^. Metallothioneins are small, cysteine-rich proteins capable of binding metal ions, the most common of which is zinc. Of the four major isoforms, the *MT1* subfamily and *MT2A* are ubiquitously expressed, whereas *MT3* and *MT4* are specific to neuronal and squamous epithelium respectively^25^. Metallothioneins bind upwards of 15% of intracellular zinc and demonstrate diverse functions including metal homoeostasis, heavy metal detoxification and protection against oxidative stress^26^. Furthermore, IFN-α and inflammatory cytokines IL-1β, IL-6 and TNF, have all been shown to induce metallothioneins, suggesting they may have a role in immunity^27^,^28^,^29^,^30^,^31^.

Here we identify zinc as the most potent inducer of metallothionein expression *in vitro*, and a strong inhibitor of IFN-λ3, but not IFN-λ1 or IFN-α signalling. Furthermore, we implicate zinc as a major driver of metallothionein expression *in vivo* and, more importantly, as an *IFNL* genotype-dependent regulator of ISGs and inflammatory cytokine expression in the livers of patients with chronic HCV infection.

## Results

### Metallothionein expression is strongly induced by zinc

**Figure 1:**
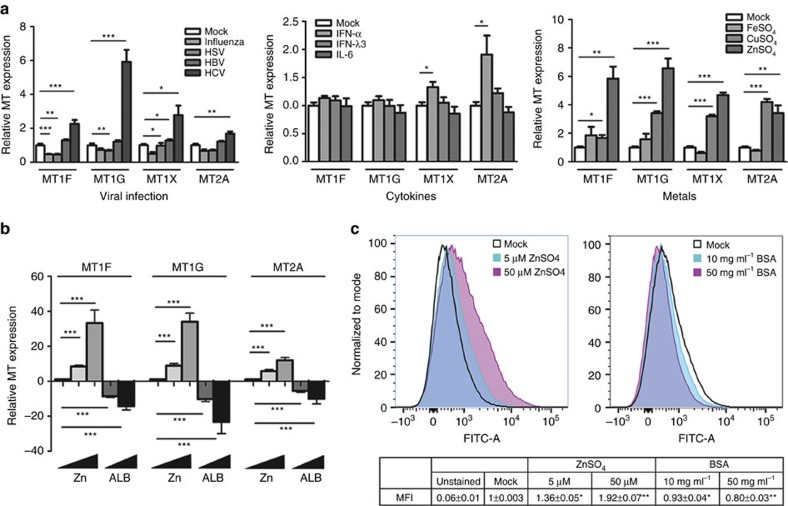
Metallothionein transcription is driven by diverse stimuli. (**a**) To examine metallothionein induction, Huh-7 cells were either virally infected, or treated with cytokines or heavy metals for 8 h. Influenza and HSV downregulated metallothionein expression whereas HBV had little effect and HCV significantly upregulated all metallothioneins. None of the cytokines examined modulated *MT1F* and *MT1G*, and only IFN-α was able to induce *MT1X* and *MT2A*. Of the metal treatments, 50 μM ZnSO_4_ most potently upregulated all the metallothioneins, followed by CuSO_4_, while FeSO_4_ had little effect (Welch's *t*-test). (**b**) Huh-7 cells treated with zinc in low serum media showed a stronger induction of metallothioneins, confirming the zinc quenching role of albumin (Welch's *t*-test). In agreement, metallothionein expression was dose-responsively reduced with BSA treatment. (**c**) Quantification of intracellular zinc by Zinpyr-1 staining confirmed that zinc and albumin dose-dependently increase and decrease intracellular zinc, respectively (Welch's *t*-test). Data are representative of three independent experiments. *P<0.05, **P<0.01, ***P<0.001, (mean±s.e.).

Metallothioneins *MT1F*, *MT1G*, *MT1H*, *MT1M* and *MT1X* are upregulated in the livers from patients with *IFN-λ rs8099917* TT responder genotypes in contrast to ISGs which are low^20^. To better understand the regulation of the metallothioneins in the liver, we examined metallothionein induction in response to three diverse stimuli: viral infection with influenza virus (H1N1), herpes simplex virus 1 (HSV-1), HBV, or HCV, cytokine treatment (IFN-α, IFN-λ3 and IL-6) and metal exposure (iron, copper and zinc). Huh-7 cells infected with HSV-1 (multiplicity of infection, (MOI)=1) or H1N1 influenza virus (MOI=0.1) demonstrated a general downregulation of metallothionein expression (Fig. 1a, left panel). However, cytopathic effect (CPE) and resulting cell death induced by both viruses was high and may have limited metallothionein induction. Conversely, HepG2 cells transfected with the HBV pUC19-HBV/E replacement replicon^32^ showed no CPE and only minor metallothionein induction (*MT1X P*<0.05, Welch's *t*-test). The only virus we assayed that significantly upregulated all metallothioneins examined, was JFH-1, a genotype 2a strain of HCV. Metallothionein expression was measured following HCV infection of Huh-7 cells (minimum 2 passages, 1 week), resulting in sustained viral replication with minimal CPE.

To confirm *IFN-λ* genotype mediates differences in metallothionein expression in an *in vitro* model of HCV infection, publicly available microarray data from primary human hepatocytes (PHHs) infected with HCV (Geo Dataset GSE54648) were used^33^. Multiple metallothioneins were significantly upregulated in PHHs with the *rs12979860* CC genotype, compared to CT/TT genotypes, in both infected hepatocytes and those adjacent to infected cells (Supplementary Fig. 1).

Unlike previous reports^27^,^34^, cytokine induction of metallothioneins in Huh-7 cells was mostly absent: treatment with IFN-α for 8 h significantly upregulated *MT1X* and *MT2A*. In contrast, IFN-λ3 and IL-6 had little effect compared to mock controls (Fig. 1a, middle panel). As expected, exposure to metal salts for 8 h induced the strongest upregulation of metallothioneins (Fig. 1a, right panel). Treatment with 50 μM ZnSO_4_ potently induced all the metallothioneins, with 100 μM CuSO_4_ evoking a weaker response, and 100 μM FeSO_4_ having no effect on metallothionein expression.

Our data are in accordance with published data demonstrating that metallothioneins are primarily induced directly by zinc and indirectly by oxidative stress (intracellular zinc release)^26^,^35^ and cytokine treatment (zinc influx)^27^,^28^,^36^,^37^. To better understand the regulation of metallothionein genes in response to zinc, we treated Huh-7 cells with ZnSO_4_ to induce metallothionein expression and bovine serum albumin (BSA) to diminish metallothionein expression. Albumin is a major carrier of zinc in the blood, and has recently been shown to potently reduce the biological effects of zinc *in vitro*^38^. To limit the zinc-quenching ability of albumin, we lowered the albumin content of the cell culture media to 1%, and found that ZnSO_4_ was far more potently able to induce expression of *MT1F* and *MT1G* in particular (Fig. 1b). Furthermore, when cells were treated with 5 μM ZnSO_4_ in increasing concentrations of albumin, a potent reduction in metallothionein expression was measured, emphasizing albumin's ability to sequester zinc and limit metallothionein expression.

To confirm these data, we measured intracellular zinc content in these cells and found that zinc and albumin dose-dependently increased or decreased intracellular zinc, respectively (Fig. 1c). Intracellular zinc content was increased almost twofold with the addition of 50 μM ZnSO_4_ (*P*<0.01, Welch's *t*-test), and subsequently reduced by over 40% with the addition of 50 mg ml^−1^ albumin (*P*<0.01, Welch's *t*-test). Lastly, by plotting intracellular zinc against metallothionein expression, we showed that *MT1F* and *MT1G* are far more responsive to intracellular zinc than *MT2A*, as demonstrated by significantly higher exponential growth rates (Supplementary Fig. 2).

### IFN-induced metallothionein expression is mediated by zinc

**Figure 2:**
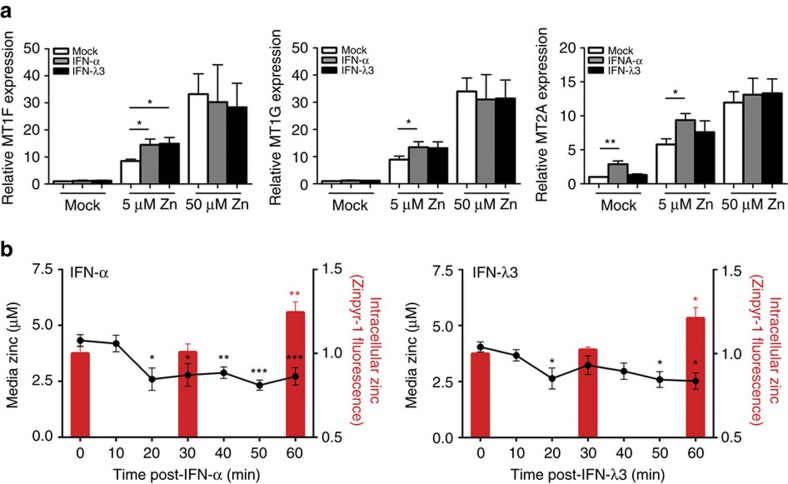
Interferon induces zinc influx and metallothionein expression in Huh-7 cells. (**a**) Huh-7 cells were with treated with either IFN-α or IFN-λ3 in media containing 1% FCS without additional zinc, 5 μM ZnSO_4_ or 50 μM ZnSO_4_. *MT1F* and *MT1G* were upregulated by interferons only in the presence of 5 μM ZnSO_4_, suggesting that their expression is mediated by zinc influx following interferon stimulation. Conversely, *MT2A* was upregulated by IFN-α even in the absence of zinc, suggesting differential regulation by interferon (Welch's *t*-test). (**b**) To confirm interferon-mediated zinc influx, cells were treated with IFN-α or IFN-λ3 and 5 μM ZnSO_4_ and intracellular/media zinc was measured using the zinc fluorophore Zinpyr-1. Both interferon treatments resulted in a zinc influx from the media into the cells, providing a mechanistic explanation for metallothionein upregulation following interferon treatment (one-way ANOVA). Data are representative of three independent experiments. *P<0.05, **P<0.01, ***P<0.001, (mean±s.e.).

To examine the relationship between IFN, zinc and subsequent metallothionein expression, Huh-7 cells were grown in media containing 1% serum and treated with either 50 U ml^−1^ IFN-α or 100 ng ml^−1^ IFN-λ3, in combination with 0, 5 and 50 μM ZnSO_4_. IFN-α-mediated *MT1F*/*MT1G* and IFN-λ3-mediated *MT1F* induction occurred only in the presence of 5 μM ZnSO_4,_ suggesting that zinc is required for interferon-mediated metallothionein expression (Fig. 2a). The addition of 50 μM zinc likely induced maximal metallothionein expression through zinc alone, preventing additional interferon-mediated metallothionein upregulation. Unlike the other metallothioneins, *MT2A* expression was induced in the absence of zinc, indicating that it may be directly regulated by interferons.

To confirm that zinc influx occurs following interferon treatment, the zinc fluorophore Zinpyr-1 was used to detect changes in intracellular and extracellular zinc in Huh-7 cell culture media treated with 5 μM ZnSO_4_ and IFN-α or IFN-λ3 over 1 h. Flow cytometry gating strategy is available in Supplementary Fig. 3. A significant reduction in media zinc was observed as early as 20 min post IFN-α or IFN-λ3 treatment, corresponding with an increase in intracellular zinc at 60 min that was only observed in interferon-treated cells (Fig. 2b). Together, these data suggest that interferon-mediated upregulation of metallothioneins *in vitro* is dependent on zinc influx and not the classical STAT1/2 signalling pathway, particularly for members of the MT1 subfamily. To confirm this hypothesis, we performed siRNA-mediated *STAT2* knockdown, and demonstrated that while IFN-λ3-induced ISG expression is drastically reduced, *MT1F* expression is not (Supplementary Fig. 4).

### Zinc inhibits the ISG response to IFN-λ3 but not IFN-α

**Figure 3:**
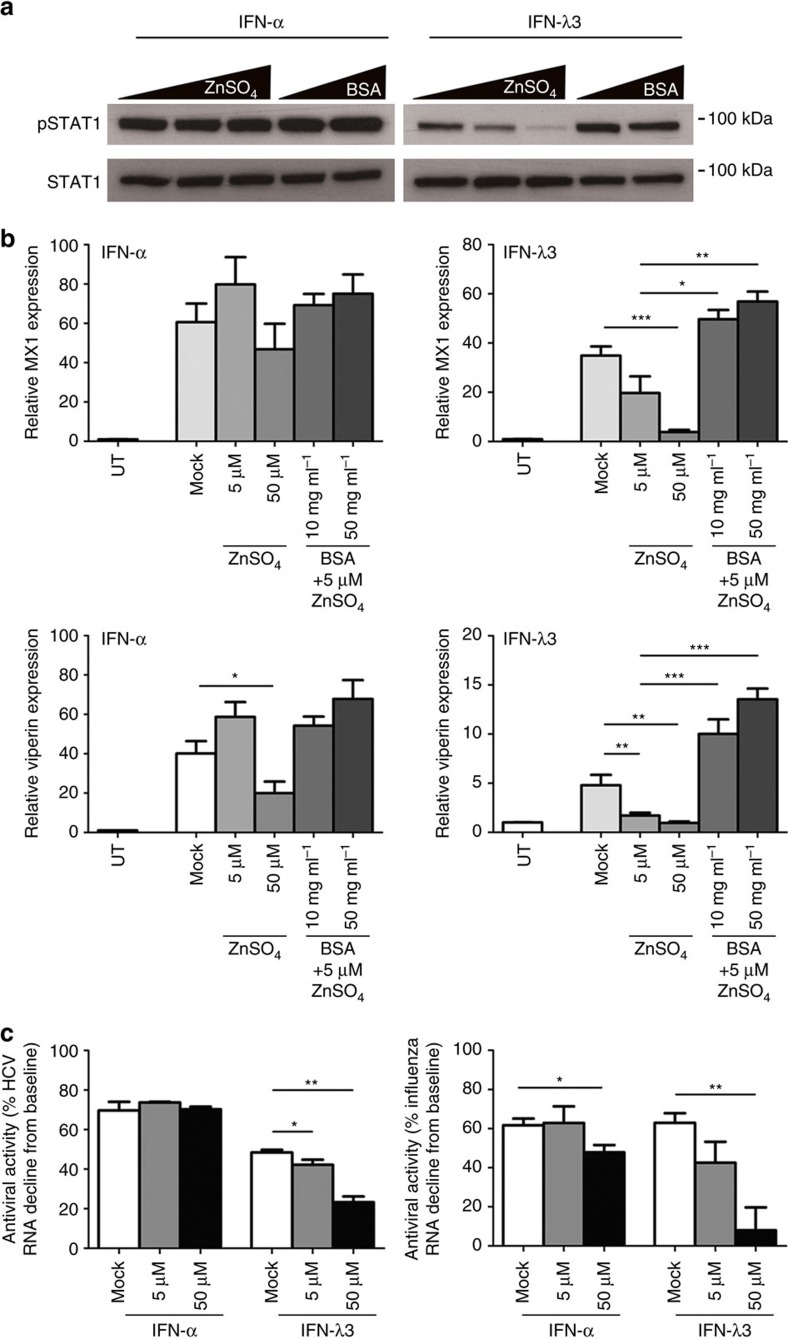
Zinc potently inhibits the IFN-λ3 response. (**a**) Huh-7 cells were treated with 50 U ml^−1^ IFN-α or 100 ng ml^−1^ IFN-λ3 in combination with increasing amounts of zinc (0, 5 and 50 μM ZnSO_4_) or 5 μM ZnSO_4_ in combination with 10 mg ml^−1^ or 50 mg ml^−1^ BSA to sequester zinc. STAT1 phosphorylation 30 min post IFN-λ3 treatment was dose-dependently inhibited by ZnSO_4_, and reversed with albumin. IFN-α treatment however, was unaffected. (**b**) The expression of IFN-λ3-induced ISGs, *MX1* and *viperin*, were affected in a similar manner, with minimal effects on IFN-α-mediated ISG expression (Welch's *t*-test). (**c**) To determine if zinc inhibits the antiviral response, Huh-7 cells infected with the JFH-1 strain of HCV and H1N1 influenza virus were treated with IFN-α and IFN-λ3 in combination with zinc. IFN-α-reduced JFH-1 intracellular RNA by ∼75% independent of zinc concentration, however the antiviral activity of IFN-λ3 was significantly reduced from ∼50% to ∼20% with 50 μM ZnSO_4_. The antiviral activity of IFN-α against influenza virus was only partially inhibited by zinc, however a similar dose-dependent inhibition of IFN-λ3 antiviral activity was observed, almost ablating its effects at 50 μM ZnSO_4_ (Welch's *t*-test). Data are representative of two (western blotting) and three (gene expression) independent experiments. *P<0.05, **P<0.01, ***P<0.001, (mean±s.e.). UT, untreated.

Zinc uptake following IFN-α or IFN-λ3 treatment suggests that intracellular zinc may influence interferon-mediated signal transduction and subsequent antiviral activity. To examine the effect of hepatic zinc on the cellular response to interferons, we modulated intracellular zinc in Huh-7 cells with ZnSO_4_ and BSA and examined downstream STAT1 activation, a signalling intermediate shared by IFN-α and IFN-λ3. Surprisingly, zinc treatment dose-dependently reduced IFN-λ3 (100 ng ml^−1^) induced STAT1 phosphorylation, but had no effect on IFN-α (50 U ml^−1^) downstream signalling (Fig. 3a). To reverse the inhibitory activity of zinc, Huh-7 cells were treated with 5 μM ZnSO_4_ in combination with albumin. IFN-λ3-mediated STAT1 phosphorylation was recovered with albumin treatment. IFN-α STAT1 phosphorylation however remained relatively unchanged. This effect was duplicated in the hepatoblastoma cell line HepG2, the colonic enterocyte cell lines Caco-2 and HCT116, and fresh peripheral blood mononuclear cells, suggesting the inhibitory effect of zinc on IFN-λ3 but not IFN-α signalling is a general phenomenon independent of the tissue or cell line studied (Supplementary Fig. 5). To ensure that our results were not due to the effect of zinc on cell viability, caspase 3/7 activation was measured, revealing no change following 24 h treatment with 50 μM ZnSO_4_ (Supplementary Fig. 6).

We next examined the expression of the IFN-responsive genes *MX1* and *viperin* to determine if reduced STAT1 activation is matched by downregulation of subsequent ISG expression. As expected, IFN-λ3-induced expression of both *MX1* and *viperin* were dose-dependently downregulated by zinc, with minimal effect on IFN-α-mediated ISG expression (Fig. 3b). Addition of albumin reversed the inhibitory effects of zinc and dose-dependently increased IFN-λ3-induced *MX1* and *viperin* expression, leaving IFN-α-induced ISG expression unchanged. Combined, these data demonstrate that zinc specifically inhibits IFN-λ3, but not IFN-α-mediated ISG induction.

### Zinc inhibits IFN-λ3, but not IFN-α antiviral activity

To test whether the inhibitory role of zinc on IFN-λ3-mediated ISG expression could translate into reduced antiviral activity, we used *in vitro* models of two different viruses, HCV-JFH-1 and influenza H1N1 virus. While IFN-α-reduced HCV intracellular RNA levels by 75% after 24 h independent of zinc concentration, the antiviral effect of IFN-λ3 was potently reduced from ∼50 to 20% by zinc (*P*<0.01, Welch's *t*-test) (Fig. 3c). Similar results were observed following H1N1 influenza infection of Huh-7 cells, as measured by extracellular influenza genome copies (<10% intracellular influenza RNA decline following IFN-α/IFN-λ3 treatment, independent of zinc). The antiviral activity of IFN-α dropped ∼20% by addition of 50 μM ZnSO_4_ (*P*<0.05, Welch's *t*-test), while the antiviral activity of IFN-λ3 was dose-dependently reduced up to 80% with the addition of zinc (*P*<0.001, Welch's *t*-test). Together, these data suggest that hepatic zinc can potently limit IFN-λ3-mediated antiviral activity not only against hepatotropic, but also other pathogenic viruses that are restricted by IFN-λ3.

### *IFNL* genotype controls serum zinc and ISG expression

**Figure 4:**
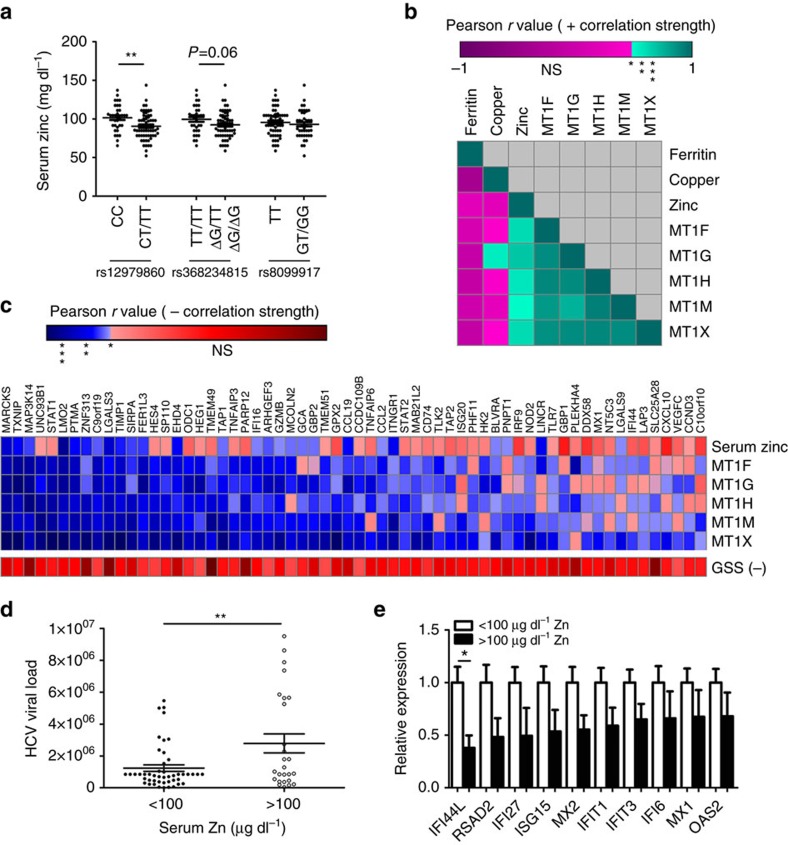
*rs12979860* CC genotype increases serum zinc whilst inhibiting hepatic ISG expression. (**a**) Significantly higher serum zinc levels were measured in chronic HCV patients that are carriers of the *IFNL* *rs12979860* CC (major) genotype (Student's *t*-test). (**b**) Serum zinc was matched to microarray expression data, and was found to significantly correlate with hepatic metallothioneins, while both serum copper and ferritin did not show any strong relationship (Pearson correlation). (**c**) Serum zinc and metallothionein expression demonstrated a strong inverse correlation with hepatic ISGs (blue—significant inverse correlation, red—NS), suggesting that zinc can inhibit ISG expression in the liver (Pearson correlation). (**d**) HCV-infected patients were categorized into low (<100 μg dl^−1^) and high (>100 μg dl^−1^) serum zinc groups, with the high zinc group possessing significantly higher viral loads (Welch's *t*-test). Anti-HCV ISG expression was lower in the high zinc group (**e**), suggesting that zinc inhibits hepatic ISG expression resulting in increased viral replication and viral load (Welch's *t*-test). *P<0.05, **P<0.01, ***P<0.001, (mean±s.e.). NS, not significant; GSS, glutathione synthetase.

The inverse relationship between metallothioneins and IFN-λ3-mediated ISG expression *in vitro* suggests that zinc may act as a regulator of IFN-λ3-mediated immunity *in vivo.* Chronic HCV infection is an optimal model to study the effects of zinc on IFN-λ3 signalling because (1) lambda interferons are the dominant antiviral interferon produced in the liver in acute HCV infection^39^ and (2) *IFN-λ* polymorphisms dictate hepatic ISG expression^19^, as well as likelihood of spontaneous and treatment induced viral clearance^1^,^2^,^3^,^4^. We previously demonstrated liver metallothionein expression was higher in HCV patients possessing the *rs12979860* CC genotype, but could not ascertain why. To determine whether zinc controls hepatic metallothionein levels in these patients, serum zinc was quantified in 100 patients with chronic HCV infection and known *rs12979860* genotype (Table 1). Serum zinc levels were significantly higher in subjects possessing the favourable *rs12979860* CC genotype as compared to CT/TT (Fig. 4a), suggesting that elevated serum zinc may drive hepatic metallothionein expression. Of note, there was no observed association between *IFN-λ4* SNP *rs368234815* and serum zinc levels despite strong linkage disequilibrium between *rs12979860* and *rs368234815* (ref. 18). A trend between serum zinc and the *IFN-λ4* SNP was observed (*P*=0.06) suggesting that our study was not sufficiently powered for this comparison, but may have reached significance with a larger sample size. To confirm the association between serum zinc and metallothionein expression, serum zinc levels were paired with metallothionein expression data obtained from our previous study^20^ and Pearson correlation was performed. Significant positive correlations were identified between serum zinc and all hepatic metallothioneins (Fig. 4b). Additionally, the expression of all the *MT1* subfamily members significantly correlated with each other (*P*<0.001, Pearson correlation), suggesting they are similarly regulated. Serum ferritin and copper showed no relationship with metallothionein expression, suggesting that zinc is the primary metal responsible for metallothionein expression in the liver.

The *IFN-λ rs12979860* CC genotype is associated with low ISG expression in the liver. Low baseline ISGs are associated with a stronger ISG response to treatment, increasing the likelihood of HCV clearance. To determine if hepatic zinc facilitates reduced ISG expression, the relationship between hepatic metallothioneins and ISGs was examined by performing Pearson correlations between the zinc responsive members of the *MT1* subfamily, MT1F/G/H/M/X, and 337 ISGs identified by Schoggins *et al*.^40^. High metallothionein expression in the liver was largely associated with low expression of most ISGs examined. To determine if the negative correlation between ISGs and metallothioneins was over-represented, a sign test was performed using a null hypothesis of 0.5 (assuming equal number of positive and negative correlations would be observed). A total of 210/337 genes demonstrated negative correlation coefficients with all metallothioneins (F, G, H, M and X), giving a highly significant *P* value (*P*<0.0001). Figure 4c depicts a heat map representation of correlation strength between each of the metallothioneins and the ISGs that showed the strongest inverse correlations. To ensure the validity of the metallothionein:ISG association, ISGs that significantly negatively correlate with at least three of five metallothioneins are listed (full list available in Supplementary Data 1). Of particular relevance, all components of the ISGF3 transcription factor complex, STAT1, STAT2 and IRF9, are present on the list. The antiviral potential of the 65 listed ISGs against numerous viruses was examined using *in vitro* ISG overexpression and infection data published by Schoggins *et al*.^40^ A total of 25/65 genes from Fig. 4c were listed as part of the top 25 antiviral genes against HCV, West Nile virus, yellow fever virus or human immunodeficiency virus HIV (Supplementary Table 1).

Pearson correlations were also performed between the ISG list and glutathione synthetase (GSS) as a negative control to rule out oxidative stress as a source of metallothionein expression/ISG suppression. GSS produces glutathione from gamma-glutamylcysteine and glycine and is a key component in antioxidant defence. GSS expression showed no relationship with hepatic metallothionein or ISG expression.

We next hypothesized that the inhibitory effect of zinc on IFN-λ3 signalling and subsequent ISG expression may increase HCV viral load. Patients were grouped into high zinc (>100 μg dl^−1^) and low zinc (<100 μg dl^−1^) subgroups and viral load was compared. Interestingly, the high zinc group demonstrated significantly higher HCV viral loads compared to the low zinc group (Fig. 4d), suggesting that zinc inhibited ISG expression may allow higher levels of viral replication. When microarray results were compared between these groups, patients with high zinc demonstrated lower expression of a number of potent antiviral ISGs (Fig. 4e). These data suggest that hepatic zinc can inhibit IFN-λ3-driven ISG expression *in vivo*, resulting in elevated viral loads. This explains a well-known, but poorly understood phenomenon, where the *rs12979860* CC genotype that confers clearance is associated with higher HCV viral loads^4^.

### Zinc modulates IFN-λ3-mediated inflammation

**Figure 5:**
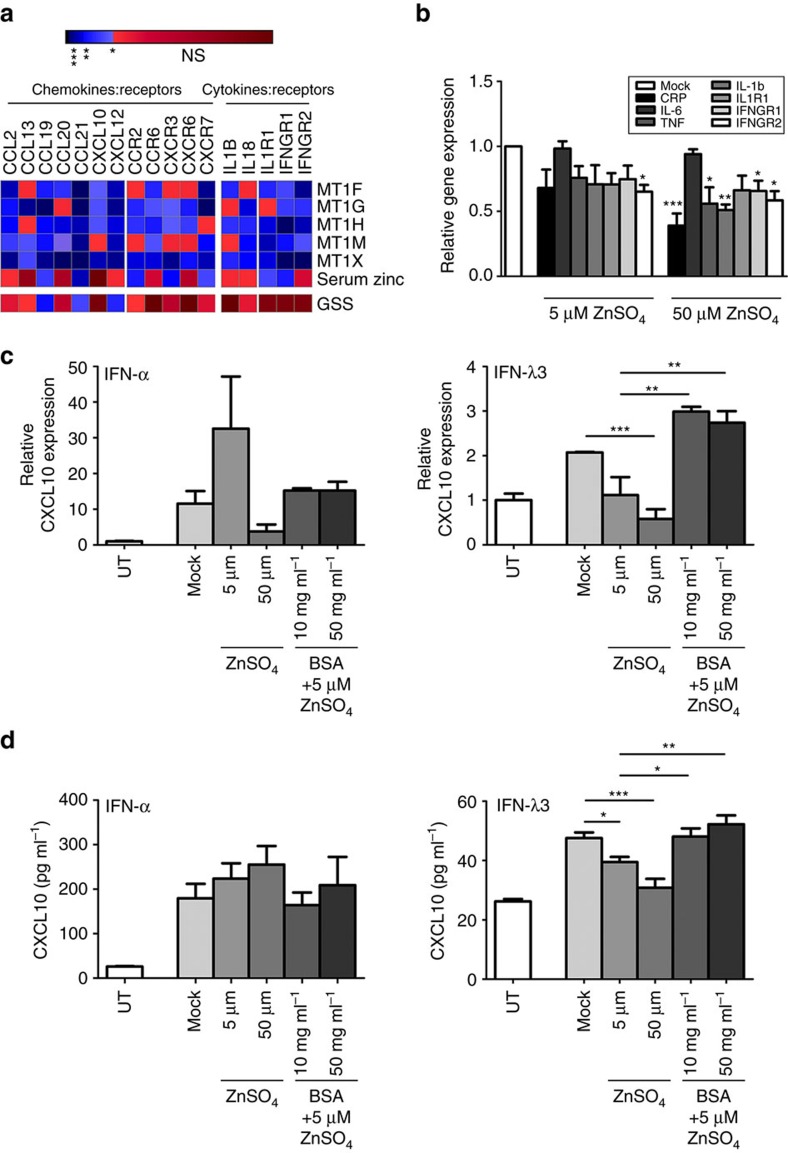
Zinc reduces hepatic chemokine and inflammatory cytokine expression. (**a**) To determine if hepatic zinc influences inflammation and chemotaxis in a clinical context, Pearson correlations were performed between biopsy metallothioneins and all measured chemokines/inflammatory cytokines and receptors. A subset of chemokines and their respective receptors both demonstrated inverse correlations with ≥3 metallothioneins, as did inflammatory cytokine receptors *IL-1R1* and *IFNGR1*/*2*. (**b**) To confirm the anti-inflammatory effects of zinc *in vitro*, JFH1 infection was used as an inflammatory stimulus in Huh-7 cells, which were treated with 5 μM and 50 μM ZnSO_4_ (Welch's *t*-test). Zinc dose-dependently reduced monocyte chemoattractant *CCL2*, inflammatory cytokines (*IL-1B*, *TNF*), receptors (*IFNGR1*/*2*) and the inflammatory marker *CRP*, particularly at the 50 μM concentration. Zinc inhibition of chemokine *CXCL10*, which is strongly expressed in response to IFN, was also measured in the presence of increasing zinc (5 and 50 μM) and albumin (10 and 50 mg ml^−1^) as performed in Fig. 3. (**c**,**d**) IFN-α-induced *CXCL10* expression showed some modulation albeit not significantly at the mRNA and protein level, contrary to IFN-λ3-mediated expression of *CXCL10*, which was dose-responsively reduced by zinc and subsequently reversed by the addition of albumin. Data are representative of three independent experiments *P<0.05, **P<0.01, ***P<0.001, (mean±s.e.). UT, untreated.

The *IFN-λ* haplotype has recently been recognized as a modulator of chronic inflammation, particularly in the liver^23^. To examine the inhibitory role of zinc on hepatic inflammation, Pearson correlations were performed between microarray measured chemokines (CC, CXC), inflammatory cytokines and their receptors, as performed in Fig. 4c. Only a fraction of genes demonstrated significant inverse correlations with >3 metallothioneins, however, those that did were chemokine:receptor combinations; *CCL2*/*CCL13*:*CCR2*, *CCL20*:*CCR6*, *CXCL10*:*CXCR3* and *CXCL12*:*CXCR7* (Fig. 5a). These data suggest hepatic zinc may also modulate chemotaxis of monocytes (CCL2), dendritic cells (CCL20), as well as T/NK cells (CXCL10) to inflamed tissues *in vivo*.

To validate the anti-inflammatory effects of zinc *in vitro,* Huh-7 cells were infected (minimum two passages, 90% infection) with the JFH-1 strain of HCV as an inflammatory stimulus, then treated with either 5 μM or 50 μM ZnSO_4_ for 24 h (Fig. 5b). Treatment with 50 μM zinc significantly reduced the expression of monocyte chemoattractant *CCL2*, inflammatory cytokines *TNF* (*P*<0.05, Welch's *t*-test), *IL-1B* (*P*<0.01, Welch's *t*-test), cytokine receptors *IFNGR1* and *IFNGR2* (*P*<0.05, Welch's *t*-test) and acute phase protein C reactive protein (*CRP*) (*P*<0.001, Welch's *t*-test) compared to mock controls. CXCL10 is an IFN-responsive chemokine that becomes greatly elevated in interferon-mediated autoimmune conditions, such as rheumatoid arthritis^41^ and chronic viral infections, such as HCV^39^,^42^. The inhibitory role of zinc on IFN-induced *CXCL10* expression was examined by treating Huh-7 cells with 50 U ml^−1^ IFN-α or 100 ng ml^−1^ IFN-λ3 and zinc/albumin similar to experiments shown in Fig. 3. IFN-λ3-mediated *CXCL10* induction was significantly reduced at both the transcript and protein level by ZnSO_4_, and was reversed by the addition of albumin (Fig. 5c). In contrast to IFN-λ3, 5 μM zinc increased IFN-α-induced *CXCL10* mRNA and protein, albeit not significantly, with variable effects at the 50 μM concentration.

### A zinc-regulated gene signature in the HCV-infected liver

A strong network of co-expression exists among the members of the *MT1* gene family that is mediated by diverse stimuli, but most potently by zinc^43^,^44^,^45^. The unique responsiveness of the metallothionein gene family to intracellular zinc provides a unique opportunity to ascertain the role of hepatic zinc on genome-wide transcription, as we have demonstrated with ISGs (Fig. 4). Spearman correlations were performed between microarray expression data from individual metallothionein genes (*MT1F*, *MT1G*, *MT1H*, *MT1M* or *MT1X*) and all other genes present on the microarray. To establish a list of genes whose expression is restricted by zinc, genes whose correlation coefficients (*r*) were positive, or negative with a *P* value >0.05 were discarded. To ensure maximum stringency, only genes that negatively correlated with all five metallothioneins were kept to form a master list of 867 genes (Supplementary Data 2).

Of these 867, a total of 798 genes were recognized by the David Bioinformatics Database^46^, and used to extract biological meaning and functional enrichment from the zinc-regulated gene set. A strong enrichment of genes mediating immunity and defence was observed, but additionally genes governing cell structure, motility, communication and adhesion were over-represented (Table 2); all biological processes that mediate immune cell trafficking to a site of inflammation. Disease classes represented by this gene set (haematological, immune and infection) support this conclusion, and suggest that zinc can regulate not only hepatic immunity (*IRF9*, *TLR7*, *STAT1*), but immune cell migration (*CXCL10*, *CCL19*, *CCL21*), adhesion (*COL1A1*, *COL3A1*, *COL4A1*, *ITGA2*, *ITGB5*) and activation (*HLA*-*A*, *HLA*-*B*, *HLA-H*).

### Zinc inhibits IFN-λ3 signalling at the receptor interface

**Figure 6:**
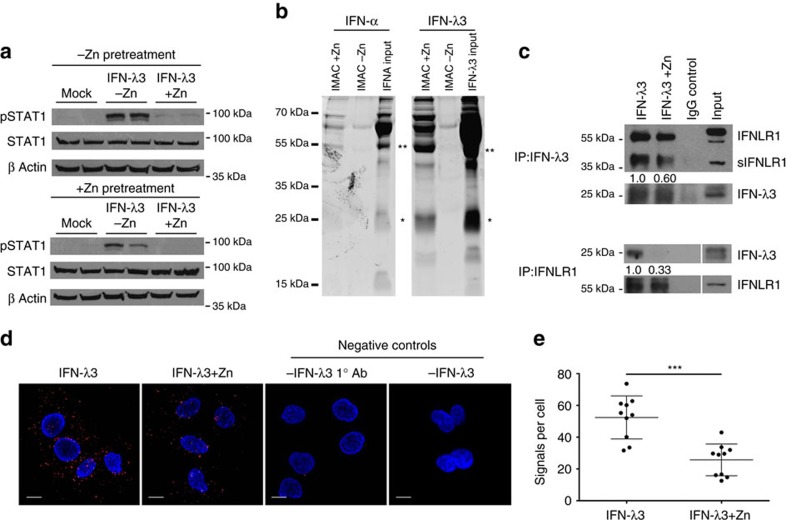
Zinc inhibits IFN-λ3:IFNLR1 binding. (**a**) To ascertain the cellular location of zinc inhibition following ZnSO_4_ treatment, Huh-7 cells were pre-treated for 24 h with 50 μM ZnSO_4_, washed thoroughly, and then treated with IFN-λ3 ±50 μM ZnSO_4_ for 15 min. STAT1 phosphorylation was significantly inhibited only when IFN-λ3 was administered in the presence of zinc, suggesting that zinc inhibits IFN-λ3 signalling at the receptor. (**b**) To identify cytokine zinc binding, 200 ng of IFN-α or IFN-λ3 were added to a zinc loaded IMAC-affinity resin for 2 h, after which bound protein was examined by SDS–PAGE. Dimeric forms of IFN-α and IFN-λ3 bound the zinc resin, as did monomeric IFN-λ3. (**c**) Using protein G beads, Co-IP was performed on Huh-7 cell lysates using recombinant IFN-λ3 alone or with the addition of 50 μM ZnSO_4_. Co-IP of both IFN-λ3 (66% reduction) and IFNLR1 (40% reduction) was reduced in the presence of zinc, when IFNLR1 and IFN-λ3 were immunoprecipitated respectively, demonstrating a direct inhibition of receptor:cytokine interaction by zinc. (**d**) Co-IP studies were confirmed using proximity ligation assays to examine the interaction of IFN-λ3 and IFNLR1 in the presence of zinc *in situ*. Confocal microscopy of IFN-λ3 treated Huh-7 cells demonstrated a reduction in fluorescent signals (red fluorescent spots), indicative of IFNLR1:IFN-λ3 interaction (within 40 nm). No signal was observed within the negative control assay lacking the primary IFN-λ3 antibody (1° Ab) or lacking the addition of IFN-λ3. (**e**) Proximity ligation signals per cell were quantified using ImageJ software, demonstrating a 50% decrease in IFNLR1:IFN-λ3 interactions per cell (Mann–Whitney test). (**d**) Scale bars, 10 μm. Data are representative of two independent experiments, ***P<0.001, (mean±s.e.).

We have demonstrated that zinc inhibits IFN-λ3 signalling, with no effect on IFN-α, suggesting that inhibition occurs at an intermediate unique to IFN-λ3. Because type I and III interferons share the ISGF3 (STAT1, STAT2, IRF9) mediated signalling pathway, there are limited possibilities to consider, apart from the IFN-λs themselves, or their receptor complex composed of IFN-λR1 and IL-10Rβ. To determine whether zinc-mediated inhibition of IFN-λ3 occurs at the receptor complex, Huh-7 cells were grown overnight in media containing 1% FBS, with or without 50 μM ZnSO_4_. Cells were washed three times, mock treated or treated with 100 ng ml^−1^ IFN-λ3, with or without 50 μM ZnSO_4_. If zinc pre-treated cells alone demonstrated inhibition of IFN-λ3 signalling, it would suggest inhibition at an intracellular location due to zinc influx over the preceding 24 h. Conversely, if inhibition occurred only when zinc was added with IFN-λ3, it would suggest an inhibition at the receptor complex. Cells were IFN-λ3 treated for 15 min and immediately lysed to examine STAT1 phosphorylation. As demonstrated in Fig. 6a, inhibition of IFN-λ3 signalling occurred only when zinc was combined with IFN-λ3 treatment, suggesting that zinc inhibits signalling at the receptor complex. Analysis of pSTAT1 by densitometry demonstrated no significant effect of zinc pre-treatment on band intensity.

To assess whether zinc inhibition of IFN-λ signalling is IFN-λ3 specific, IFN-λ1-mediated STAT1 phosphorylation in Huh-7 and HepG2 cells was examined in the presence of zinc (Supplementary Fig. 7). Interestingly, no inhibition of IFN-λ1-induced STAT1 phosphorylation was observed with zinc treatment, suggesting that zinc likely hinders the interaction between IFN-λ3 and its receptor. To investigate the capacity of IFN-λ3 to bind zinc, 200 ng of recombinant IFN-λ3 or IFN-α were loaded onto IMAC-select affinity resin charged with or without Zn^2+^ ions. Following a 2 h incubation, proteins were removed from the resin with EDTA, run on a 10% polyacrylamide gel and stained with SYPRO ruby protein stain. Ultraviolet visualization of eluted protein demonstrated that IFN-λ3 bound the zinc resin in monomeric (*) and dimeric (**) forms, whereas IFN-α bound predominantly as a dimer (Fig. 6b), supporting previous findings^47^.

To confirm that zinc can specifically inhibit the interaction between IFN-λ3 and IFN-λR1, co-immunoprecipitation (co-IP) experiments were performed. Huh-7 cell lysates were combined with recombinant IFN-λ3 alone or with 50 μM ZnSO_4_, and immunoprecipitated with protein G beads and antibodies for both IFN-λR1 or IFN-λ3. Figure 6c demonstrates that zinc inhibits IFN-λR1 binding to IFN-λ3 following co-IP of IFN-λR1 with IFN-λ3 (40% reduction in binding) and co-IP of IFN-λ3 with IFN-λR1 (66% reduction in binding), as quantified by densitometry. Moreover, our data is in agreement with the publication by Witte *et al*. demonstrating an interaction between soluble IFN-λR1 (sIFN-λR1) and IFN-λ1 (ref. 48).

Co-IP experiments were next validated using the Duolink proximity ligation assay with IFN-λR1 and IFN-λ3 specific antibodies. Proximity ligation applies probes to species-specific antibodies that hybridize if they are within 40 nm of each other, become amplified by a rolling circle method, and to which a fluorescent probe can be bound. Compared to IFN-λ3 alone, the addition of 50 μM ZnSO_4_ to IFN-λ3 treatment (100 ng ml^−1^) significantly reduced the number of fluorescent signals per cell, representing interactions between IFN-λR1 and IFN-λ3 (Fig. 6d). Interaction signals per cell dropped from ∼52±4.27 to 26±3.18 (*P*<0.001, Mann–Whitney test) by the addition of ZnSO_4_ (Fig. 6e). Negative control proximity ligation assay (PLA) experiments omitting either IFN-λ3 or the IFN-λ3 antibody (Fig. 6d) demonstrated no amplified signal, ensuring the validity of the assay. Additional PLAs were performed on Jurkat and K562 cell lines lacking *IFN-λR1* expression, and resulted in no amplified signal, indicative of IFN-λR1:IFN-λ3 interaction (Supplementary Fig. 8).

A unique structural difference between IFN-λ1 and IFN-λ3 is their disulphide bond pattern^49^,^50^. We hypothesized that zinc may interfere with a disulphide bond that is present in IFNλ-2/3, but not IFN-λ1 or IFN-λ4 (refs 49, 50). This hypothesis was based on the well documented zinc inhibition of disulphide bond formation^51^,^52^. Mass spectrometry studies performed however, demonstrated no effect of zinc on IFN-λ3 disulphide bond status (Supplementary Note, Supplementary Table 2 or 3).

### IFN-λ3 drives zinc movement across intestinal epithelium

**Figure 7:**
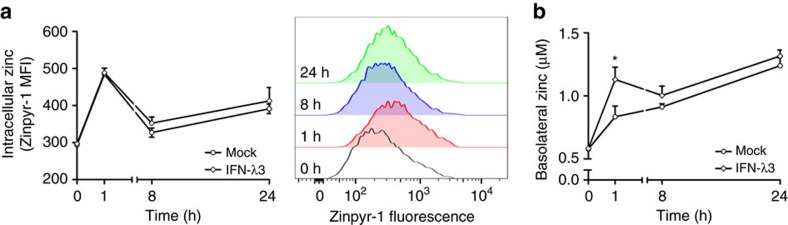
IFN-λ3 drives intestinal zinc transport *in vitro.* To examine the role of IFN-λ3 on intestinal zinc absorption, a transwell zinc absorption assay was performed using a differentiated Caco-2 monolayer. Caco-2 cells were treated with 5 μM ZnSO_4_ with or without IFN-λ3 and zinc was measured both intracellularly and across the monolayer in the lower (basolateral) chamber. (**a**) Intracellular zinc was increased independent of IFN-λ3 treatment, peaking at 1 h (*P<0.05) but remaining elevated until 24 h post treatment. (**b**) IFN-λ3 increased basolateral zinc over the course of 24 h, reaching significance at 1 h and remaining elevated for the remainder of the experiment. Data are representative of two independent experiments, *P<0.05, (mean±s.e.).

Because the *rs12979860* CC genotype has been shown to mediate higher hepatic IFN-λ3 expression^53^,^54^,^55^, we hypothesized that elevated serum IFN-λ3 could drive zinc absorption across the intestinal epithelium; an effect observed during endotoxemia^56^. To examine the movement of zinc across a monolayer *in vitro*, a transwell assay measuring IFN-λ3-mediated movement of zinc across a Caco-2 monolayer was performed. Caco-2 cells are colonic epithelial cells that, once differentiated over 3 weeks, form microvilli and tight junctions, mimicking the intestinal epithelium^57^. Following 3 weeks of culture, monolayer integrity was examined by performing a fluorescein permeability assay, achieving <3% permeability indicative of true monolayer formation (Supplementary Fig. 8). Cell monolayers were then mock treated or treated with IFN-λ3 in combination with 5 μM ZnSO_4_, and intracellular zinc content as well as basolateral (lower chamber) zinc concentration were measured using the zinc fluorophore Zinpyr-1. An intermediate concentration of 5 μM ZnSO_4_ was used to measure zinc movement across the monolayer without fully inhibiting IFN-λ3 signalling, as demonstrated in Supplementary Fig. 5. Figure 7a demonstrates that treatment with 5 μM ZnSO_4_ increased intracellular zinc content irrespective of IFN-λ3, over 24 h. In contrast, basolateral zinc concentrations significantly increased at 1 h following IFN-λ3 treatment and remained elevated compared to mock control, albeit not significantly (Fig. 7b). Together, these data suggest IFN-λ3 can drive zinc uptake and transport across the intestinal epithelium, and provide a mechanism for elevated serum zinc levels in patients possessing the *rs12979860* responder (CC) genotype.

## Discussion

**Figure 8:**
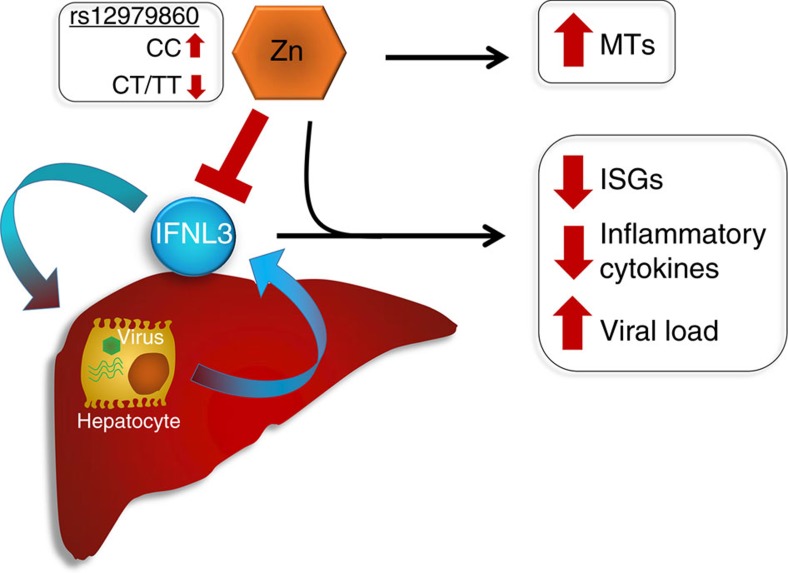
Inhibition of IFN-λ3 signalling by zinc. Elevated hepatic zinc drives metallothionein expression whilst inhibiting IFN-λ3. Inhibition of IFN-λ3 reduces ISG and inflammatory gene expression resulting in increased viral load, a common feature in patients possessing the favourable *IFNL* *rs12979860* CC genotype.

This study identifies for the first time that zinc is a potent, specific inhibitor of IFN-λ3 signalling. We also demonstrate *in vitro* that zinc is a potent driver of metallothionein expression and is linked to type I/III interferon response by influx. Zinc inhibited IFN-λ3 signalling *in vitro* by interfering with IFN-λ3:IFN-λR1 signalling at the receptor, but demonstrated little effect on the IFN-α response. We found a strong negative association between hepatic metallothionein and ISG/chemokine expression in patients with chronic HCV, suggesting that zinc mediates induction of metallothioneins *in vivo*, while inhibiting IFN-λ3-mediated ISG expression (Fig. 4). Furthermore, serum zinc was higher in HCV-infected patients possessing the *rs12979860* CC genotype, suggesting it may drive down pre-treatment ISG expression to facilitate a strong immune response (Fig. 8). By creating a master list of genes that show a significant inverse correlation with all quantified *MT1* genes, we created a list of potential zinc-regulated genes with roles relating to the immune response, cell motility and adhesion.

Zinc is an acute phase reactant that rapidly relocates from the blood into the liver, infected tissue and peripheral blood mononuclear cell populations following infection and cytokine stimulation^58^. Subsequently, it has been difficult to distinguish the source of metallothionein induction *in vivo*, as zinc relocation is an integral part of systemic immunity. We found that metallothionein induction was most responsive to zinc treatment, but was also upregulated in response to HCV infection *in vitro*. Acute HCV infection induces a significant amount of oxidative stress within infected hepatocytes^59^, which is a key driver of metallothionein expression due to the release of intracellular zinc stores^26^. HCV induced inflammatory cytokine expression however, likely plays a small role in metallothionein induction following infection, as we demonstrated only mild metallothionein expression following interferon/IL-6 treatment. Interestingly, chronic HCV infection *in vivo* has been shown to reduce plasma zinc as well as hepatic metallothionein expression, suggesting chronic infection/inflammatory cytokine exposure has a markedly different effect on zinc distribution than the acute response^60^.

In our experiments, interferon stimulation induced a modest increase in expression of *MT1* subfamily genes that was zinc-influx dependent as has been previously shown^28^. Although we demonstrated zinc inhibition of IFN-λ3 signalling (Fig. 3), the concentration used (5 μM ZnSO_4_) was not sufficient to fully inhibit the interaction between IFN-λ3 and IFN-λR1, but was sufficient to demonstrate zinc influx in response to interferons. Unlike the *MT1* subfamily, *MT2A* was upregulated by IFN-α even in the absence of zinc, suggesting that it is a classical STAT1:STAT2 dependent ISG. Using the UCSC genome browser^61^, analysis of experimentally validated transcription factor binding to *MT1* subfamily versus *MT2A* promoter regions substantiates this hypothesis (Supplementary Fig. 10). Inflammatory transcription factor binding within the promoters of *MT1* subfamily members is limited and likely mediated primarily by zinc, while *MT2A* demonstrates an accumulation of STAT1, IRF1, STAT3, NFκB and AP-1 binding sites.

The role of zinc in the interferon response has been widely studied, but remains poorly understood. In persons with zinc deficiency, zinc supplementation improves not only the type I and II interferon production/response^62^,^63^, but also immune cell survival, maturation and function^64^,^65^. Because up to 10% of the human proteome is capable of binding zinc^66^, narrowing down zinc-specific mechanisms of immune activation/repression has been difficult, and is indeed multifactorial^67^. We demonstrated an improvement in the IFN-α response in the presence of 5 μM ZnSO_4_, resulting in a moderate increase in *MX1*, *viperin* and *CXCL10* expression. Zinc-mediated IFN-α dimers have been purified by crystallography^47^, and have been demonstrated in our zinc binding experiments (Fig. 6b), however the resulting effect on antiviral activity remains unknown.

Our novel finding that zinc can specifically inhibit IFN-λ3 signalling, suggests interference upstream of STAT1 phosphorylation, which most likely occurs at the receptor binding interface. Pre-treatment with zinc (Fig. 6a) demonstrated no major effect on IFN-λ3-induced STAT1 phosphorylation when cells were subsequently washed and zinc was not included with the cytokine. These data suggest that intracellular zinc does not play a significant role in blocking IFN-λ3 signalling, and rules out an interaction between zinc and STAT1, or other intracellular IFN-signalling intermediates as a possible mechanism of inhibition. Potent inhibition of IFN-λ3 signalling occurred only when zinc was included with IFN-λ3, indicating that it likely interferes with the interferon:receptor interaction. In addition, zinc was unable to prevent IFN-λ1 signalling (Supplementary Fig. 6), which binds to a similar IFN-λR1 region as IFN-λ3 (refs 49, 50), and suggests that zinc binding to IFN-λ3 may specifically inhibit receptor interaction. We subsequently confirmed zinc binding to IFN-λ3 as well as inhibition of receptor binding by both co-IP and proximity ligation assay (Fig. 6), strongly supporting this hypothesis. Based on the data however, it is difficult to completely exclude an interaction of zinc with IFN-λR1 that affects only the interaction with IFN-λ3, as both IFN-λ3 and IFN-λ1 have different tertiary structures and thus may interact differently with IFN-λR1 residues^49^,^50^. Further studies are required to clarify the mechanistic model of zinc inhibition, and whether it is indeed an interaction between zinc and IFN-λ3. Circular dichroism spectroscopy to determine if zinc can alter IFN-λ3 tertiary structure or mutational analysis of IFN-λ3/IFN-λR1 to identify the specific residues through which zinc interferes with IFN-λ3 signalling can be considered to elucidate this complex mechanism.

The inverse relationship observed between ISG expression and metallothioneins/serum zinc suggests IFN-λs are the major mediators of ISG expression in HCV-infected liver. Indeed, this has been shown in experimental models of acute HCV infection in chimpanzees and PHHs^39^,^68^. Furthermore, elevated serum zinc and hepatic metallothionein expression in patients with the *rs12979860* CC genotype provides a mechanism to explain their reduced expression of hepatic ISGs prior to treatment. Low baseline ISG expression in the liver of patients with HCV is beneficial as it provides a basis for a potent antiviral response in the context of IFN-α based therapy^69^, but this may not be the case for other viral infections controlled by IFN-λ3, and requires further study^7^,^9^. The mechanism of elevated serum zinc in patients with the *IFN-λ rs12979860* CC genotype may be mediated by increased circulating IFN-λ3, which we have shown can drive intestinal zinc uptake *in vitro* (Fig. 7). These data indicate that elevated serum IFN-λ3 in these individuals may increase serum zinc, which can become reduced following chronic HCV infection^60^, thereby facilitating a stronger hepatic immune response. Our group and others have demonstrated a strong association between *IFN-λ* polymorphisms and chronic inflammation in chronic viral and non-viral liver disease, suggesting that zinc supplementation may provide a useful, well tolerated adjunct to modulate inflammation.

The strong regulatory role of zinc on ISG expression led us to believe that it may mediate similar immune repression on a genome-wide scale. By identifying only genes that showed significant inverse correlation with all metallothioneins, we achieved a more stringent identification of 867 genes that are most likely to be regulated by intracellular zinc (Supplementary Data 2). Because this list only includes genes that correlate with all five metallothioneins, we likely excluded a number of zinc-regulated genes, including a number of ISGs, cytokines and chemokines we identified in Figs 4 and 5. Nonetheless, the gene list was created to elucidate biological processes that are most likely regulated by zinc, and confirm that metallothionein expression reflects tissue zinc in our model. Concentrated within this gene set were biological processes related not only to innate immunity, but monocyte/DC/NK/T cell chemo-attraction and adhesion (Table 2). Zinc inhibition of the intracellular inflammatory response has been shown previously^70^,^71^, but our data suggest a further inhibition of immune cell recruitment and activation at sites of inflammation. In concordance, we demonstrated a strong zinc-mediated inhibition of IFN-λ3-induced *CXCL10* mRNA and protein expression *in vitro*. This adds another layer of complexity to zinc biology, as zinc has classically been shown to improve immune cell function^64^, but our data suggest it may also inhibit recruitment to inflamed tissue.

In summary, we have demonstrated for the first time that IFN-λ3 signalling is directly inhibited by zinc. Elevated hepatic zinc, as suggested by increased metallothionein expression, mediates a reduction in ISG expression in patients with the *rs12979860* CC genotype, thus facilitating viral clearance following treatment. Furthermore, using the co-expressed MT1 gene family as a marker for hepatic zinc, we have demonstrated that zinc inhibits numerous facets of the hepatic immune response that may be mediated by IFN-λ3, including leukocyte chemotaxis and adhesion. These studies support the potential utility of zinc as a simple and effective treatment to modulate IFN-λ3-mediated immune activation in human disease.

## Methods

### Study cohort

All samples were collected from untreated HCV genotype 1 infected patients. The zinc study cohort consists of the pre-treatment serum samples from 100 patients taken at the first clinical visit. Parallel liver function and clinical blood tests were performed by the Institute for Clinical Pathology and Medical Research (ICPMR) at Westmead Hospital, Sydney. The microarray sample cohort consists of 22 patients liver biopsies^20^, 17 of which belong to the zinc study cohort. Briefly, liver biopsy RNA was isolated using the Qiagen RNeasy kit and mRNA was hybridized to Illumina Human HT-12 V3 arrays according to the manufacturers instructions. Signal intensity was measured using Beadstudio version 3 and cubic spline normalization was applied. Microarray data is available in the Dryad repository under doi:10.5061/dryad.n0s75. Ethics approval was obtained from the Sydney West Area Health Service and University of Sydney. All subjects have given written formal consent (HREC2002/12/4.9(1564)). All subjects were genotyped for the *IFN-λ* SNP *rs12979860* using Taqman genotyping probes (ThermoFisher)^20^.

### Serum and media zinc quantification

De-proteinized serum zinc was quantified by a colorimetric assay using the Sentinel zinc quantification kit (17640H) by ICPMR at Westmead Hospital, Sydney. Huh-7 media zinc was quantified by measuring Zinpyr-1 fluorescence at 530 nm using the VICTOR plate reader and a standard curve generated with ZnSO_4_.

### Cell culture and heavy metal/cytokine treatments

Huh-7 and HepG2 cell lines were cultured in Dulbecco's minimal essential medium (DMEM) with 10% foetal bovine serum (FBS) unless otherwise stated. Individual ZnSO_4_, CuSO_4_ and FeSO_4_ (Sigma Aldrich), IFN-α (Roferon-A), IFN-λ1 and IFN-λ3 (R&D Systems) treatments were all performed for 8 h to determine mRNA transcript expression or 24 h for protein quantitation (ELISA), unless stated otherwise. Prior to interferon treatments, zinc/albumin pre-treatment was performed overnight, and IFN-α/IFN-λ was subsequently added the next day in fresh media containing zinc/albumin.

### Flow cytometry

Huh-7 cells were cultured for 24 h in media containing ZnSO_4_ and BSA, trypsinized, washed twice in PBS and stained for 15 min at room temperature with 5 μM Zinpyr-1 (Santa-Cruz) and LIVE/DEAD cell stain (Life Technologies). Cells were then washed twice with cold PBS and fluorescence was examined using the Canto II/Fortessa flow cytometer (BD Bioscience). Cells were gated for individual cells lacking fluorescence under the ultraviolet filter 450/50 (live), and zinc fluorescence was measured with the 520/20 filter. Geometric mean and median fluorescence were measured for each treatment and compared to mock treatments to examine alterations in intracellular zinc content. Rapid detection of zinc influx following cytokine treatment was performed similarly on Huh-7 cells, however concurrent administration of interferon and Zinpyr-1 was necessary to ensure timely zinc quantification.

### Viral infections

Active replication of the genotype 2a JFH-1 strain^72^ of HCV was initiated by viral RNA electroporation into Huh-7 cells. HCV RNA from the pJFH1 plasmid was transcribed *in vitro* using the T7 RiboMAX express large-scale RNA production system (Promega) and transfected into Huh-7 cells. Approximately 1–2 weeks post electroporation, HCV infectivity was measured by qPCR and immunofluorescence using an anti-NS5A antibody (kindly provided by Prof. Mark Harris, University of Leeds, UK) as in ref. 73 and RNA was extracted.

To establish HBV replication, the HBV pUC19-HBV/E replacement replicon^32^, which achieves higher replication and hepatitis B core antigen (HbcAg) levels than wild type, was transfected into HepG2 cells using Fugene HD (Promega) according to the manufacturer's protocol. Three days post infection, HBcAg from culture media was measured using the QuickTiter HBV core antigen ELISA Kit (Cell Biolabs), HBcAg was detected by immunofluorescence using an anti-HBcAg antibody (Dako) to ensure adequate infection and RNA was extracted for metallothionein quantification.

Influenza virus infection (grown and titered in MDCK cells) was achieved using the H1N1 A/California/01/2009 strain (kindly provided by Dr Ken McPhie of ICPMR at Westmead Hospital) in Huh-7 cells. Briefly, Huh7 cells were infected at an MOI of 0.1 in R-Mix Refeed medium (Diagnostic Hybrids Inc.) for 1 h at 33 °C, being centrifuged at 3,000 r.p.m. for the first 30 min. After 1 h, an equal volume of DMEM plus 20% FBS was added to the cells, and infection was allowed to proceed for another 23 h. Virus was removed after a total of 24 h, and cells were collected after 72 h (at the peak of intracellular viral RNA levels) for quantification of intracellular influenza RNA and metallothionein quantification.

HSV-1 strain F was grown and titered in Vero cells (kindly provided by Dr Negar Talaei Zanjani of the Centre for Virus Research, Westmead Millennium Institute for Medical Research, Sydney). To prevent cytokine contamination from growth media, virus was purified by high speed centrifugation using a sucrose cushion gradient^24^. Huh-7 cells were infected at an MOI of 1, and RNA was harvested 24 h after infection.

### Quantitative PCR

RNA extractions were performed using the Favorgen Tissue Total RNA kit according to the manufacturer's protocol, and cDNA was synthesized out of 500 ng of RNA with MMLV reverse transcriptase (Promega). Transcript quantification was performed using the Rotor-gene 6000 thermocycler (Corbett Research) with the following TaqMan primer probes from Applied Biosystems: *18s* (4319413E), *MT1F* (Hs00744661_sH), *MT1G* (Hs02578922_gH), *MT1X* (Hs00745167_sH), *MT2A* (Hs02379661_g1), *CXCL10* (Hs01124251_g1), HCV 5′UTR (Pa03453408_s1). Primer sets used with Fast SYBR Mastermix (Life Technologies) are as follows: *CCL2* (CTGCTCATAGCAGCCACCTT, GCACTGAGATCTTCCTATTGGTG), *CCL19* (GCCTGCTGGTTCTCTGGAC, GCATGGGTTTCTGGGTCAC), *CXCL12* (GCAATGCCATCATCTCCTG, CGAAGCACAGGTGACACG), *CRP* (AGCTGGGAGTCCGCCTCAGG, AGGACTGAAGGGCCCGCCAA), *IL-1B* (TCGCCAGTGAAATGATGGCT, GGTCGGAGATTCGTAGCTGG), *IL-1R* (TTGCGTGGTAAGAAATTCATCTT, TGCTTAAATATGGCTTGTGCATT), *IFNGR1* (GGATTCCAGTTGTTGCTGCT, CACAGAGATCAAGGACTTGGGTA), *IFNGR2* (AAATACACCGACAGTAAATGGTTCA, GCGGCAGTGAAGTCACACT), *TNF* (CCCGAGTGACAAGCCTGTAG, TGAGGTACAGGCCCTCTGAT), *IL-6* (GAGGGAGCCTGGCCTTCGGA, AGCACTGGGGTGGGTCGAGG) and influenza *M* gene (AATGCCCTAAATGGGAATGG, GGCAAGTGCACCAGTTGAAT). Standard curves using fivefold cDNA dilutions were used to determine relative gene expression, and all mRNA levels were normalized using 18 s ribosomal RNA.

### Protein quantitation by western blotting and ELISA

Following ZnSO_4_ and BSA treatment, Huh-7 cells were lysed, and subject to SDS–polyacrylamide gel electrophoresis. Protein was transferred on to nitrocellulose membrane, and β-actin and STAT1 were probed using STAT1 (Santa Cruz Biotechnology, SC-345 clone C-24, rabbit anti-human, 1:2,000 dilution), Tyr701 p-STAT1 (Cell Signalling, 9167 clone 58D6, rabbit anti-human, 1:500 dilution) and β-actin (Sigma-Aldrich, A1978 clone AC-15, mouse anti-human, 1:10,000 dilution) antibodies. Full blots are available in Supplementary Fig. 11. CXCL10 ELISA (BioLegend) was performed on cell culture supernatant from IFN, zinc and albumin treated Huh-7 cells according to the manufacturer's instructions.

### Zinc binding assays

To examine the ability of IFN-α or IFN-λ3 to bind zinc, IMAC-Select affinity gel (Sigma-Aldrich) was used according to the manufacturer's instructions. Briefly, IMAC gel was incubated with 50 mM ZnSO_4_ for 1 h at 4 °C, washed thoroughly with equilibration buffer (50 mM NaH_2_PO_4_, 300 mM NaCl, pH 8.0) and incubated with 200 ng of IFN-α or IFN-λ3 at 4 °C for 1 h. IMAC gel was then thoroughly washed and bound protein was eluted using 100 mM EDTA. Eluted protein was separated by SDS–PAGE, stained with SYPRO ruby (Thermo Fisher Scientific), and visualized using a ultraviolet transilluminator (Bio-Rad).

### Co-immunoprecipitation assays

Huh-7 cells scraped from 150 cm^2^ flasks were incubated for 10 min in IP lysis buffer (150 mM NaCl, 50 mM Tris-HCl pH 7.4, 1% NP-40, 0.25% deoxycholate, 1 mM EGTA, 1 mM PMSF, 1 mM sodium orthovanadate, 1 mM sodium fluoride) and passed through a 21 Gauge needle ten times to ensure sample homogenization. Recombinant human IFN-λ3 (100 ng ml^−1^) was added to the cell lysates either alone, or in combination with ZnSO_4_ to a final concentration of 50 μM. Lysates were pre-cleared with recombinant protein G sepharose beads (Life Technologies) for 1 h at 4 °C to prevent non-specific binding, and incubated with either an anti-IFN-λ3 (RnD Systems, MAB5259 clone #567143, mouse anti-human) diluted 1:100 or anti-IFN-λR1 antibody (Sigma Aldrich, HPA017319, rabbit anti-human) diluted 1:100 overnight at 4 °C. Protein G beads were next added to cell lysates and incubated at 4 °C for 2 h. Beads were spun down, washed four times in PBS to remove any unbound protein, and incubated in Western blot loading buffer containing SDS and 2-mercaptoethanol to dissociate protein-bead complexes. Western blots were performed as stated above using IFN-λR1 (Sigma Aldrich, HPA017319, rabbit anti-human) and IFN-λ3 (RnD Systems, MAB5259 clone #567143, mouse anti-human) antibodies diluted 1:1,000.

### Proximity ligation assay

Huh-7 cells cultured on glass coverslips were treated for 10 min with 100 ng ml^−1^ IFN-λ3 alone or with 50 μM ZnSO_4_. Cells were fixed with 4% PFA for 10 min, blocked with 2% fetal calf serum (FCS) and incubated with both IFN-λR1 (Sigma Aldrich, HPA017319, rabbit anti-human, diluted 1:100) and IFN-λ3 (RnD Systems, MAB5259 clone #567143, mouse anti-human, diluted 1:100) specific antibodies overnight at 4 °C. The Duolink proximity ligation assay (Sigma Aldrich) was performed to identify IFN-λR1:IFN-λ3 interactions *in situ* according to the manufacturer's instructions. Cells were visualized using the Olympus FV1000 confocal microscope and signals were identified and counted using ImageJ software. For each treatment, ten fields of view were imaged, signals counted and normalized to the number of cells in the field. Over 50 cells were counted for each treatment.

### Cysteine residue modification analysis

IFN-λ3 protein (R&D Systems) was analysed under three conditions; untreated with alkylation using 10 mM IAA for 30 min at 37 °C, zinc-treated (10 mM) for 30 min at 37 °C with alkylation or dithiothreitol-treated (10 mM) for 30 min at 56 °C. All samples were trypsin digested and peptides were separated by nanoLC using an Ultimate nanoRSLC UPLC and autosampler system (Dionex, Amsterdam, Netherlands). Samples (2.5 μl) were concentrated and desalted onto a micro C18 precolumn (300 μm × 5 mm, Dionex) with H_2_O:CH_3_CN (98:2, 0.1% TFA) at 15 μl min^−1^. After a 4 min wash, the pre-column was switched (Valco 10 port UPLC valve, Valco, Houston, TX) into line with a fritless nano column (75 μm × ∼15 cm) containing C18AQ media (1.9 μm, 120 Å Dr Maisch, Ammerbuch-Entringen Germany). Peptides were eluted using a linear gradient of H_2_O:CH_3_CN (98:2, 0.1% formic acid) to H_2_O:CH_3_CN (64:36, 0.1% formic acid) at 200 nl min^−1^ over 30 min. High voltage (2,000 V) was applied to low volume Titanium union (Valco) with the column oven heated to 45 °C (Sonation, Biberach, Germany) and the tip positioned ∼0.5 cm from the heated capillary (*T*=300 °C) of a QExactive Plus (Thermo Electron, Bremen, Germany) mass spectrometer. Positive ions were generated by electrospray and the QExactive operated in a data dependent acquisition mode.

A survey scan *m*/*z* 350–1,750 was acquired (resolution=70,000 at *m*/*z* 200, with an accumulation target value of 1,000,000 ions) and lockmass enabled (*m/z* 445.12003). Up to the ten most abundant ions (>80,000 counts, underfill ratio 10%) with charge states >+2 and <+7 were sequentially isolated (width *m*/*z* 2.5) and fragmented by HCD (NCE=30) with a AGC target of 100,000 ions (resolution=17,500 at *m*/*z* 200). *m*/*z* Ratios selected for mass spectrometry (MS)/MS were dynamically excluded for 30 s. Peak lists were generated using Mascot Daemon/Mascot Distiller (Matrix Science, London, England) or Proteome Discoverer (Thermo, v1.4) using default parameters, and submitted to the database search programme Mascot (version 2.5.1, Matrix Science). Search parameters were: Precursor tolerance 4 p.p.m. and product ion tolerances±0.05 Da; Met(O) carboxyamidomethyl-Cys specified as variable modification, enzyme specificity was trypsin, 1 missed cleavage was possible and the non-redundant protein database from NCBI (January 2015) searched.

### Redox state analysis

The redox state of the IFN-λ3 disulphide bonds were determined using differential cysteine alkylation and MS^74^. Briefly, unpaired cysteine thiols in IFN-λ3 were alkylated with 5 mM 2-iodo-*N*-phenylacetamide (^12^C-IPA, Cambridge Isotopes) and the protein resolved on reducing SDS–PAGE and stained with Coomassie (Sigma). The IFN-λ3 band was excised, destained, dried, incubated with 40 mM dithiothreitol and washed. The fully reduced protein was alkylated with 5 mM 2-iodo-*N*-phenylacetamide where all six carbon atoms of the phenyl ring have a mass of 13 (^13^C-IPA, Cambridge Isotopes). The gel slice was washed and dried before digestion with 12 ng μl^−1^ of trypsin (Roche). Peptides were eluted from the slices, resolved by liquid chromatography and analysed by MS as described^74^. The redox state of the disulphide bond cysteines was calculated from the relative ion abundance of peptides encompassing one or both disulphide cysteines labelled with ^12^C-IPA (reduced) or ^13^C-IPA (oxidized). Peptides containing the underlined disulphide cysteines were resolved and analysed: Cys188-Cys195, DLNCVASGDLCVHHHHHH; Cys37-Cys126, GCHIAQFK; Cys71-Cys169, ESPGCLEASVTFN-λFR. To calculate ion abundance of peptides, extracted ion chromatograms were generated using the XCalibur Qual Browser software (v2.1.0; Thermo Scientific). The area was calculated using the automated peak detection function built into the software. The ratio of ^13^C-IPA to ^12^C-IPA+^13^C-IPA labelled peptides represents the fraction of the cysteine in the population that is in the oxidized state.

### Transcellular zinc transport across gut epithelium *in vitro*

To investigate intestinal zinc transport in response to IFN-λ3 treatment, monolayers of Caco-2 cells were grown on polyethylene terephthalate transwell inserts. Briefly, 1.5 × 10^5^cells per cm^2^ were allowed to differentiate for 25 days at 37 °C with 5% CO_2_ to form a confluent monolayer and best simulate the gastrointestinal epithelium. To assure a fully formed monolayer, a fluorescein permeability assay was performed for each experiment. PBS containing 0.01% fluorescein sodium salt (cell impermeable) was added to the transwell insert and incubated at 37 °C for 1 h. After incubation, 0.01% fluorescein (starting concentration) and aliquots of PBS from the bottom well were read at 530 nm using the VICTOR plate to determine monolayer permeability. Confluent monolayers were determined by obtaining a value of <3% using the equation (% permeability=100 × (bottom well fluorescence/0.01% fluorescein fluorescence)).

To investigate IFN-λ3-mediated zinc transport, Caco-2 monolayers grown overnight in media containing 1% FBS and 5 μM ZnSO_4_ were treated with fresh media containing 1% FBS and 5 μM ZnSO_4_±100 ng ml^−1^ IFN-λ3. An intermediate zinc concentration of 5 μM was used to determine the effects of IFN-λ3 on zinc transport, without fully inhibiting IFN-λ3 signalling which was measured in Caco-2 cells at 50 μM ZnSO_4_ (Supplementary Fig. 5). At 1 and 8 h post treatment, media from both the upper and lower chambers, and Caco-2 cells were collected to measure zinc transport and uptake respectively. Zinpyr-1 was used to quantify both cellular and media zinc using fluorescence assays as described above.

### Statistical analysis

GraphPad Prism 5 was used to compare *in vitro* treatments and patient serum concentration using the Student's *t*-test, Welch's *t*-test and one-way ANOVA (as indicated). Pearson correlation was used to examine the relationship between biopsy microarray gene signatures and serum zinc. Correlation between serum zinc, MT1s identified as upregulated in subjects possessing the *IFN-λ* responder genotype by O'Connor *et al*.^20^ and antiviral effector ISGs identified by Schoggins *et al*. were performed^40^. Metallothionein:ISG correlations included in this manuscript demonstrate significant inverse correlation between any single ISG and at least three of five metallothioneins (F, G, H, M, X). A complete list can be obtained in Supplementary Material.

### Data availability

The data that support the findings of this study has been uploaded into the Dryad repository under the digital object identifier doi:10.5061/dryad.n0s75. Additional relevant data are available from the authors upon reasonable requests.

## Additional information

**How to cite this article:** Read, S. A. *et al*. Zinc is a potent and specific inhibitor of IFN-λ3 signalling. *Nat. Commun.* **8**, 15245 doi: 10.1038/ncomms15245 (2017).

**Publisher's note:** Springer Nature remains neutral with regard to jurisdictional claims in published maps and institutional affiliations.

## Supplementary Material

Supplementary Information Supplementary Figures, Supplementary Tables, Supplementary Note and Supplementary References

Supplementary Data 1 Interferon stimulated genes that negatively correlate with MT1s and serum zinc.

Supplementary Data 2 Full list of genes that inversely correlate with 5 hepatic metallothioneins.

## Figures and Tables

**Table 1:** Patient characteristics stratified by IFNL *rs12979860* genotype.

**rs12979860**	**All**	**CC**	**CT/TT**	***P*** **value**
*Patient characteristics*				
*N*	100	35	65	
Age (year)	46.13±1.31	44.82±2.21	46.81±1.63	NS
Male/female	61/39	20/15	41/24	NS
Aspartate aminotransferase (IU l^−1^)	80.90±5.54	95.26±11.70	73.29±5.62	NS
Alanine aminotransferase (IU l^−1^)	111.20±9.54	144.80±23.35	93.36±7.00	0.009
Gamma-glutamyltranspeptidase (IU l^−1^)	103.40±9.30	83.07±13.18	113.8±13.18	NS
Total bilirubin (mg dl^−1^)	13.36±0.68	11.97±0.82	13.90±0.80	NS
Albumin (g dl^−1^)	4.14±0.04	4.17±0.06	4.13±0.06	NS
Ferritin (ng ml^−1^)	302.70±39.35	311.1±86.11	298.2±39.58	NS
Haemoglobin (g l^−1^)	148.00±2.16	145.6±3.21	149.2±2.84	NS
Copper (μg dl^−1^)	111.00±4.95	114.7±4.68	109.6±6.62	NS
Zinc (μg dl^−1^)	94.47±1.75	101.7±2.85	90.66±2.07	0.003
Viral load (× 10^3^ copies)	2,134±279	3,318±65	1,542±234	0.002

NS, not significant.

Data represented as mean ± s.e.m.

**Table 2:** Zinc-regulated transcriptome disease class and biological process as identified by DAVID functional annotation.

	***P*** **value**	**Genes**	**Fold enrichment**	**Example genes**
*Disease class*				
Cardiovascular	0.014	59/798	1.3	*TIMP1*, *PTGDS*, *TNFAIP3*, *GPX3*
Immune	0.016	67/798	1.3	*IRF8, CXCL12, HLA-A, JUND*
Vision	0.018	21/798	1.7	*C7, FBLN5, LAMC1, PARP1*
Haemotological	0.024	17/798	1.8	*G6PD, ITGAV, IL7R, PROCR*
Infection	0.083	27/798	1.4	*DDX5, STAT1, TLR7, GBP2*
				
*Panther biological process*				
Cell structure and motility	5.20E−10	94/798	1.9	*COL1A1*, *ITGB5*, *JAG1*, *RHOA*
Immunity and defense	1.60E−07	97/798	1.7	*IRF9*, *HLA-B*, *NFKB1*, *TAP1*
Cell communication	1.40E−04	80/798	1.5	*CD40LG, CCL19, CCL2, CXCL10*
Cell adhesion	1.80E−04	47/798	1.8	*CD34, ITGA2, COL14A1, VWF*
Signal transduction	3.30E−04	187/798	1.2	*RAB11A, FOXO3, IFNGR1, MAPK15*

## References

[b1] Thomas D. L. . Genetic variation in IL28B and spontaneous clearance of hepatitis C virus. Nature 461, 798–801 (2009). 19759533 10.1038/nature08463 PMC3172006

[b2] Suppiah V. . IL28B is associated with response to chronic hepatitis C interferon-alpha and ribavirin therapy. Nat. Genet. 41, 1100–1104 (2009). 19749758 10.1038/ng.447

[b3] Tanaka Y. . Genome-wide association of IL28B with response to pegylated interferon-alpha and ribavirin therapy for chronic hepatitis C. Nat. Genet. 41, 1105–1109 (2009). 19749757 10.1038/ng.449

[b4] Ge D. . Genetic variation in IL28B predicts hepatitis C treatment-induced viral clearance. Nature 461, 399–401 (2009). 19684573 10.1038/nature08309

[b5] Patel D. A. . Interferon response and respiratory virus control are preserved in bronchial epithelial cells in asthma. J. Allergy Clin. Immunol. 134, 1402–1412.e1407 (2014). 25216987 10.1016/j.jaci.2014.07.013 PMC4261010

[b6] Egli A. . IL-28B is a key regulator of B- and T-cell vaccine responses against influenza. PLoS Pathog. 10, e1004556 (2014). 25503988 10.1371/journal.ppat.1004556 PMC4263767

[b7] Pott J. . IFN-lambda determines the intestinal epithelial antiviral host defense. Proc. Natl Acad. Sci. USA 108, 7944–7949 (2011). 21518880 10.1073/pnas.1100552108 PMC3093475

[b8] Baldridge M. T. . Commensal microbes and interferon-lambda determine persistence of enteric murine norovirus infection. Science 347, 266–269 (2015). 25431490 10.1126/science.1258025 PMC4409937

[b9] Jewell N. A. . Lambda interferon is the predominant interferon induced by influenza A virus infection *in vivo*. J. Virol. 84, 11515–11522 (2010). 20739515 10.1128/JVI.01703-09 PMC2953143

[b10] Bullens D. M. . Type III IFN-lambda mRNA expression in sputum of adult and school-aged asthmatics. Clin. Exp. Allergy 38, 1459–1467 (2008). 18564328 10.1111/j.1365-2222.2008.03045.x

[b11] Nice T. J. . Interferon-lambda cures persistent murine norovirus infection in the absence of adaptive immunity. Science 347, 269–273 (2015). 25431489 10.1126/science.1258100 PMC4398891

[b12] Wu Q. . Serum IFN-lambda1 is abnormally elevated in rheumatoid arthritis patients. Autoimmunity 46, 40–43 (2013). 23039206 10.3109/08916934.2012.730587

[b13] Wu Q., Yang Q., Lourenco E., Sun H. & Zhang Y. Interferon-lambda1 induces peripheral blood mononuclear cell-derived chemokines secretion in patients with systemic lupus erythematosus: its correlation with disease activity. Arthritis Res. Ther. 13, R88 (2011). 21679442 10.1186/ar3363 PMC3218903

[b14] Sommereyns C., Paul S., Staeheli P. & Michiels T. IFN-lambda (IFN-lambda) is expressed in a tissue-dependent fashion and primarily acts on epithelial cells *in vivo*. PLoS Pathog. 4, e1000017 (2008). 18369468 10.1371/journal.ppat.1000017 PMC2265414

[b15] Bolen C. R., Ding S., Robek M. D. & Kleinstein S. H. Dynamic expression profiling of type I and type III interferon-stimulated hepatocytes reveals a stable hierarchy of gene expression. Hepatology 59, 1262–1272 (2014). 23929627 10.1002/hep.26657 PMC3938553

[b16] Zhou Z. . Type III interferon (IFN) induces a type I IFN-like response in a restricted subset of cells through signaling pathways involving both the Jak-STAT pathway and the mitogen-activated protein kinases. J. Virol. 81, 7749–7758 (2007). 17507495 10.1128/JVI.02438-06 PMC1933366

[b17] Ding S. & Robek M. D. Peroxisomal MAVS activates IRF1-mediated IFN-lambda production. Nat. Immunol. 15, 700–701 (2014). 25045870 10.1038/ni.2924

[b18] Prokunina-Olsson L. . A variant upstream of IFN-λ3 (IL28B) creating a new interferon gene IFN-λ4 is associated with impaired clearance of hepatitis C virus. Nat. Genet. 45, 164–171 (2013). 23291588 10.1038/ng.2521 PMC3793390

[b19] Urban T. J. . IL28B genotype is associated with differential expression of intrahepatic interferon-stimulated genes in patients with chronic hepatitis C. Hepatology 52, 1888–1896 (2010). 20931559 10.1002/hep.23912 PMC3653303

[b20] O'Connor K. S. . Hepatic metallothionein expression in chronic hepatitis C virus infection is IFN-λ3 genotype-dependent. Genes Immun. 15, 88–94 (2014). 24335707 10.1038/gene.2013.66

[b21] Galmozzi E. & Aghemo A. Nonsynonymous variant Pro70Ser (rs117648444) in IFN-λ4 gene identifies carriers of the rs368234815 DeltaG allele with higher HCV RNA decline during the first 4 weeks of pegylated interferon and ribavirin therapy in HCV-1 patients. J. Clin. Virol. 59, 274–275 (2014). 24495847 10.1016/j.jcv.2014.01.006

[b22] Sugiyama M., Tanaka Y., Wakita T., Nakanishi M. & Mizokami M. Genetic variation of the IL-28B promoter affecting gene expression. PLoS ONE 6, e26620 (2011). 22046316 10.1371/journal.pone.0026620 PMC3201970

[b23] Eslam M. . Interferon-lambda rs12979860 genotype and liver fibrosis in viral and non-viral chronic liver disease. Nat. Commun. 6, 6422 (2015). 25740255 10.1038/ncomms7422 PMC4366528

[b24] Talaei Zanjani N. . Abalone hemocyanin blocks the entry of Herpes Simplex Virus 1 into cells: a potential new antiviral strategy. Antimicrob. Agents Chemother. 60, 1003–1012 (2015). 26643336 10.1128/AAC.01738-15 PMC4750698

[b25] Babula P. . Mammalian metallothioneins: properties and functions. Metallomics 4, 739–750 (2012). 22791193 10.1039/c2mt20081c

[b26] Zhang B. . Activity of metal-responsive transcription factor 1 by toxic heavy metals and H_2_O_2_ *in vitro* is modulated by metallothionein. Mol. Cell Biol. 23, 8471–8485 (2003). 14612393 10.1128/MCB.23.23.8471-8485.2003 PMC262672

[b27] Nagamine T. . Interferonbeta-induced changes in metallothionein expression and subcellular distribution of zinc in HepG2 cells. Cytokine 34, 312–319 (2006). 16884910 10.1016/j.cyto.2006.06.008

[b28] Sato M. . Metallothionein synthesis induced by interferon alpha/beta in mice of various zinc status. Tohoku J. Exp. Med. 178, 241–250 (1996). 8727706 10.1620/tjem.178.241

[b29] Schroeder J. J. & Cousins R. J. Interleukin 6 regulates metallothionein gene expression and zinc metabolism in hepatocyte monolayer cultures. Proc. Natl Acad. Sci. USA 87, 3137–3141 (1990). 2326272 10.1073/pnas.87.8.3137 PMC53849

[b30] Cousins R. J. & Leinart A. S. Tissue-specific regulation of zinc metabolism and metallothionein genes by interleukin 1. FASEB J. 2, 2884–2890 (1988). 2458983 10.1096/fasebj.2.13.2458983

[b31] Sato M., Sasaki M. & Hojo H. Differential induction of metallothionein synthesis by interleukin-6 and tumor necrosis factor-alpha in rat tissues. Int. J. Immunopharmacol. 16, 187–195 (1994). 8181907 10.1016/0192-0561(94)90075-2

[b32] Fujiwara K. . Novel type of hepatitis B virus mutation: replacement mutation involving a hepatocyte nuclear factor 1 binding site tandem repeat in chronic hepatitis B virus genotype E. J. Virol. 79, 14404–14410 (2005). 16254374 10.1128/JVI.79.22.14404-14410.2005 PMC1280239

[b33] Sheahan T. . Interferon lambda alleles predict innate antiviral immune responses and hepatitis C virus permissiveness. Cell Host Microbe 15, 190–202 (2014). 24528865 10.1016/j.chom.2014.01.007 PMC4104123

[b34] Guevara-Ortiz J. M., Omar-Castellanos V., Leon-Chavez B. A., Achanzar W. E. & Brambila E. Interferon alpha induction of metallothionein in rat liver is not linked to interleukin-1, interleukin-6, or tumor necrosis factor alpha. Exp. Mol. Pathol. 79, 33–38 (2005). 16005709 10.1016/j.yexmp.2005.02.005

[b35] Dalton T. P., Li Q., Bittel D., Liang L. & Andrews G. K. Oxidative stress activates metal-responsive transcription factor-1 binding activity. Occupancy *in vivo* of metal response elements in the metallothionein-I gene promoter. J. Biol. Chem. 271, 26233–26241 (1996). 8824273 10.1074/jbc.271.42.26233

[b36] Brieger A., Rink L. & Haase H. Differential regulation of TLR-dependent MyD88 and TRIF signaling pathways by free zinc ions. J. Immunol. 191, 1808–1817 (2013). 23863901 10.4049/jimmunol.1301261

[b37] Liuzzi J. P. . Interleukin-6 regulates the zinc transporter Zip14 in liver and contributes to the hypozincemia of the acute-phase response. Proc. Natl Acad. Sci. USA 102, 6843–6848 (2005). 15863613 10.1073/pnas.0502257102 PMC1100791

[b38] Haase H., Hebel S., Engelhardt G. & Rink L. The biochemical effects of extracellular Zn and other metal ions are severely affected by their speciation in cell culture media. Metallomics 7, 102–111 (2014). 25360687 10.1039/c4mt00206g

[b39] Thomas E. . HCV infection induces a unique hepatic innate immune response associated with robust production of type III interferons. Gastroenterology 142, 978–988 (2012). 22248663 10.1053/j.gastro.2011.12.055 PMC3435150

[b40] Schoggins J. W. . A diverse range of gene products are effectors of the type I interferon antiviral response. Nature 472, 481–485 (2011). 21478870 10.1038/nature09907 PMC3409588

[b41] Lee E. Y., Lee Z. H. & Song Y. W. The interaction between CXCL10 and cytokines in chronic inflammatory arthritis. Autoimmun. Rev. 12, 554–557 (2013). 23092582 10.1016/j.autrev.2012.10.001

[b42] Brownell J. . Independent, parallel pathways to CXCL10 induction in HCV-infected hepatocytes. J. Hepatol. 59, 701–708 (2013). 23770038 10.1016/j.jhep.2013.06.001 PMC3779522

[b43] Choi J. K., Yu U., Yoo O. J. & Kim S. Differential coexpression analysis using microarray data and its application to human cancer. Bioinformatics 21, 4348–4355 (2005). 16234317 10.1093/bioinformatics/bti722

[b44] Prieto C., Risueno A., Fontanillo C. & De las Rivas J. Human gene coexpression landscape: confident network derived from tissue transcriptomic profiles. PLoS ONE 3, e3911 (2008). 19081792 10.1371/journal.pone.0003911 PMC2597745

[b45] Chen C. . Two gene co-expression modules differentiate psychotics and controls. Mol. Psychiatry 18, 1308–1314 (2013). 23147385 10.1038/mp.2012.146 PMC4018461

[b46] Huang, da W., Sherman B. T. & Lempicki R. A. Systematic and integrative analysis of large gene lists using DAVID bioinformatics resources. Nat. Protoc. 4, 44–57 (2009). 19131956 10.1038/nprot.2008.211

[b47] Radhakrishnan R. . Zinc mediated dimer of human interferon-alpha 2b revealed by X-ray crystallography. Structure 4, 1453–1463 (1996). 8994971 10.1016/s0969-2126(96)00152-9

[b48] Witte K. . Despite IFN-lambda receptor expression, blood immune cells, but not keratinocytes or melanocytes, have an impaired response to type III interferons: implications for therapeutic applications of these cytokines. Genes Immun. 10, 702–714 (2009). 19798076 10.1038/gene.2009.72

[b49] Gad H. H. . Interferon-lambda is functionally an interferon but structurally related to the interleukin-10 family. J. Biol. Chem. 284, 20869–20875 (2009). 19457860 10.1074/jbc.M109.002923 PMC2742852

[b50] Miknis Z. J. . Crystal structure of human interferon-lambda1 in complex with its high-affinity receptor interferon-lambdaR1. J. Mol. Biol. 404, 650–664 (2010). 20934432 10.1016/j.jmb.2010.09.068 PMC2991516

[b51] Jakob U., Muse W., Eser M. & Bardwell J. C. Chaperone activity with a redox switch. Cell 96, 341–352 (1999). 10025400 10.1016/s0092-8674(00)80547-4

[b52] Briggs D. B. . Zinc enhances adiponectin oligomerization to octadecamers but decreases the rate of disulfide bond formation. Biometals 25, 469–486 (2012). 22234497 10.1007/s10534-012-9519-9

[b53] Dill M. T. . Interferon-induced gene expression is a stronger predictor of treatment response than IL28B genotype in patients with hepatitis C. Gastroenterology 140, 1021–1031 (2011). 21111740 10.1053/j.gastro.2010.11.039

[b54] Shi X. . IL28B genetic variation is associated with spontaneous clearance of hepatitis C virus, treatment response, serum IL-28B levels in Chinese population. PLoS ONE 7, e37054 (2012). 22649509 10.1371/journal.pone.0037054 PMC3359351

[b55] Rallon N. I. . Impact of IL28B gene polymorphisms on interferon-lambda3 plasma levels during pegylated interferon-alpha/ribavirin therapy for chronic hepatitis C in patients coinfected with HIV. J. Antimicrob. Chemother. 67, 1246–1249 (2012). 22294646 10.1093/jac/dkr598 PMC3695611

[b56] Pekarek R. S. & Evans G. W. Effect of acute infection and endotoxemia on zinc absorption in the rat. Proc. Soc. Exp. Biol. Med. 150, 755–758 (1975). 1108043 10.3181/00379727-150-39119

[b57] Rousset M. The human colon carcinoma cell lines HT-29 and Caco-2: two *in vitro* models for the study of intestinal differentiation. Biochimie 68, 1035–1040 (1986). 3096381 10.1016/s0300-9084(86)80177-8

[b58] Haase H. & Rink L. Zinc signals and immune function. Biofactors 40, 27–40 (2014). 23804522 10.1002/biof.1114

[b59] Lin W. . Hepatitis C virus regulates transforming growth factor beta1 production through the generation of reactive oxygen species in a nuclear factor kappaB-dependent manner. Gastroenterology 138, 2509–2518 (2010). 20230822 10.1053/j.gastro.2010.03.008 PMC2883661

[b60] Nagamine T. . The possible role of zinc and metallothionein in the liver on the therapeutic effect of IFN-alpha to hepatitis C patients. Biol. Trace Elem. Res. 58, 65–76 (1997). 9363321 10.1007/BF02910667

[b61] Kent W. J. . The human genome browser at UCSC. Genome Res. 12, 996–1006 (2002). 12045153 10.1101/gr.229102 PMC186604

[b62] Cakman I., Kirchner H. & Rink L. Zinc supplementation reconstitutes the production of interferon-alpha by leukocytes from elderly persons. J. Interferon Cytokine Res. 17, 469–472 (1997). 9282827 10.1089/jir.1997.17.469

[b63] Prasad A. S. Effects of zinc deficiency on Th1 and Th2 cytokine shifts. J. Infect. Dis. 182, S62–S68 (2000). 10944485 10.1086/315916

[b64] Prasad A. S. Zinc: role in immunity, oxidative stress and chronic inflammation. Curr. Opin. Clin. Nutr. Metab. Care 12, 646–652 (2009). 19710611 10.1097/MCO.0b013e3283312956

[b65] Prasad A. S. Zinc in human health: effect of zinc on immune cells. Mol. Med. 14, 353–357 (2008). 18385818 10.2119/2008-00033.Prasad PMC2277319

[b66] Andreini C., Banci L., Bertini I. & Rosato A. Counting the zinc-proteins encoded in the human genome. J Proteome Res. 5, 196–201 (2006). 16396512 10.1021/pr050361j

[b67] Berg J. M. & Shi Y. The galvanization of biology: a growing appreciation for the roles of zinc. Science 271, 1081–1085 (1996). 8599083 10.1126/science.271.5252.1081

[b68] Park H. . IL-29 is the dominant type III interferon produced by hepatocytes during acute hepatitis C virus infection. Hepatology 56, 2060–2070 (2012). 22706965 10.1002/hep.25897 PMC3581145

[b69] Sarasin-Filipowicz M. . Interferon signaling and treatment outcome in chronic hepatitis C. Proc. Natl Acad. Sci. USA 105, 7034–7039 (2008). 18467494 10.1073/pnas.0707882105 PMC2383932

[b70] Liu M. J. . ZIP8 regulates host defense through zinc-mediated inhibition of NF-kappaB. Cell Rep. 3, 386–400 (2013). 23403290 10.1016/j.celrep.2013.01.009 PMC3615478

[b71] Zhou Z. . Abrogation of nuclear factor-kappaB activation is involved in zinc inhibition of lipopolysaccharide-induced tumor necrosis factor-alpha production and liver injury. Am. J. Pathol. 164, 1547–1556 (2004). 15111301 10.1016/s0002-9440(10)63713-3 PMC1615672

[b72] Wakita T. . Production of infectious hepatitis C virus in tissue culture from a cloned viral genome. Nat. Med. 11, 791–796 (2005). 15951748 10.1038/nm1268 PMC2918402

[b73] Read S. A., Tay E., Shahidi M., George J. & Douglas M. W. Hepatitis C virus infection mediates cholesteryl ester synthesis to facilitate infectious particle production. J. Gen. Virol. 95, 1900–1910 (2014). 24859394 10.1099/vir.0.065300-0

[b74] Bekendam R. H. . A substrate-driven allosteric switch that enhances PDI catalytic activity. Nat. Commun. 7, 12579 (2016). 27573496 10.1038/ncomms12579 PMC5013553

