# Supplementary material for: Zinc is a potent and specific inhibitor of IFN-λ3 signalling
Source: Nat Commun. 2017 May 17;8:15245. doi: 10.1038/ncomms15245 (PMC5442324; doi:10.1038/ncomms15245)
Supplement: Supplementary Information — Supplementary Figures, Supplementary Tables, Supplementary Note and Supplementary References [file ncomms15245-s1.pdf]

Supplementary Note 1

Because the inhibition of disulphide bond formation by zinc is well documented [1-3], we hypothesized that zinc may interfere with disulphide bonds unique to IFNL3.

To characterize the potential for zinc to disrupt the IFNL3:IFNLR1 receptor interface by inhibiting the disulphide bond unique to IFNL3, two mass spectrometry studies were performed: 1) Recombinant IFNL3 protein was mock treated, zinc treated, or fully reduced using DTT, followed by alkylation with iodoacetamide (IAA). Reduced cysteines lacking disulfide bonds become carbamidomethylated (CAM) with IAA, whereas oxidized cysteines that maintain their disulfide bond do not (dehydro). 2) The redox state of all disulphide bonds was measured using differential cysteine alkylation (^{12}C and ^{13}C) in the native protein, zinc-treated or EDTA-treated IFNL3 protein.

In the first experimental setting, we hypothesized that zinc would successfully stabilise the reduced form of the unique disulphide belonging to IFNL3 composed of cysteines 188 and 195, resulting in CAM modifications on those residues. Conversely, DTT would reduce all disulphide bonds resulting in CAM modifications of all residues. Over several experiments, zinc treatment of IFNL3 did not reduce this disulphide bond, which displayed an oxidised stable bond, similar to the untreated IFNL3 protein (Supplementary Table 4). In contrast, in DTT-treated, alkylated IFNL3, all cysteine resides were CAM modified, indicating successful reduction of the disulphide bonds. Interestingly, while the mock and zinc treated C188-195 disulphide bond appeared to be stably oxidised in these analyses (as shown by the presence of 2 Dehydro modifications), the C169 residue was CAM modified, indicating reduction of the C71-C169 disulphide bond (Supplementary Table 4).

When we undertook the second approach, the redox state of the three disulphide bonds in IFNL3 (Cys188-Cys195, Cys71-Cys169 and Cys37-Cys136; UniProt numbering) were determined by differential cysteine alkylation. This was done by labelling free/reduced

cysteine residues with ^{12}C, followed by DTT-treatment to reduce all remaining disulphide bonds. Lastly, ^{13}C labelling and mass spectrometry was performed [4]. Both the Cys188-Cys195 and Cys37-Cys136 disulphide bonds were 97-100% oxidised in the protein, while the Cys71-Cys169 was again confirmed to exist in both reduced (7-10%) and oxidised (90-93%) forms (Supplementary Table 5). Incubation of IFNL3 with zinc ions (10 mM) or EDTA (10 mM) to remove endogenous metal ions from the protein did not change the redox state of the disulphide bonds. This finding indicates that the unique Cys188 and Cys195 resides in IFNL3 are disulphide bonded and do not coordinate zinc ions. Importantly, the finding that the C169 residue exists in the native protein as a combination of both reduced and oxidized disulphides bonded to C71 is in stark contrast to the reported crystal structure [5], which demonstrates a static structure containing oxidized, stable disulphide bonds at the C71-169 site. Therefore, the reported crystal structure for IFNL3 limits the success of modelling potential zinc-binding sites. Indeed, when we conducted tertiary structure searches using four different algorithms for analysis of structure files to identify zinc binding, no zinc binding sites were identified (Zinc binding tools; http://ligin.weizmann.ac.il/~lpgerzon/mbs4/mbs3.cgi [6], http://bioinf.cs.ucl.ac.uk/structure/ [7], http://netalign.ustc.edu.cn/temsp/index.php [8], http://feature.stanford.edu/metals/ [9]. This does not however indicate there are no zinc binding sites in IFNL3, as the zinc binding assay in Fig.6B clearly shows IFNL3 binds to zinc. Further, this does not indicate there are no zinc binding sites specifically at the IFNL3:IFNLR1 receptor interface, as our Co-IP data in Fig.6C clearly demonstrates a biochemical interaction between IFNL3 and IFNLR1, which is inhibited by zinc. The analysis does however suggest that the currently available crystal structure of IFNL3 does not allow identification of dynamic ligand binding sites capable of coordinating a zinc atom. This would require additional crystal structures, ideally of IFNL3 complexed to IFNLR1, and

extensive mutational analysis of all potential resides involved in the receptor interface to help identify essential residues for zinc binding.

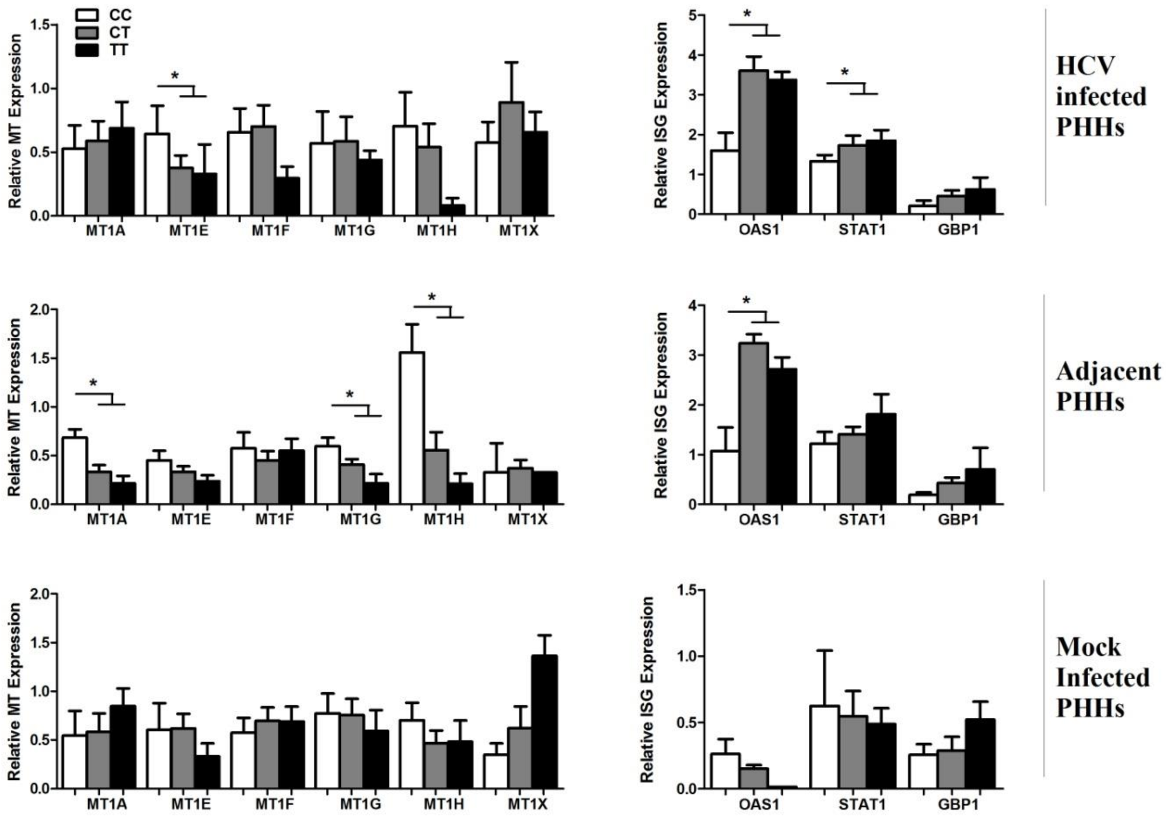

Supplementary Figure 1. IFNL genotype dependent MT expression in primary human hepatocytes.

Differential gene expression between HCV infected primary human hepatocytes (PHHs), adjacent uninfected cells, and mock infected cells, performed using laser capture microdissection [10] was examined in the publicly available Geo Dataset GSE54648. MT expression was significantly elevated in HCV infected and adjacent hepatocytes from individuals possessing the *rs12979860* CC allele compared to the CT/TT alleles. This trend was not observed in mock infected cells, suggesting that viral infection and subsequent immune activation drives IFNL3 genotype dependent MT expression. Data are representative of 5-13 samples. Welch's t test, * $p<0.05$, (mean ± SE).

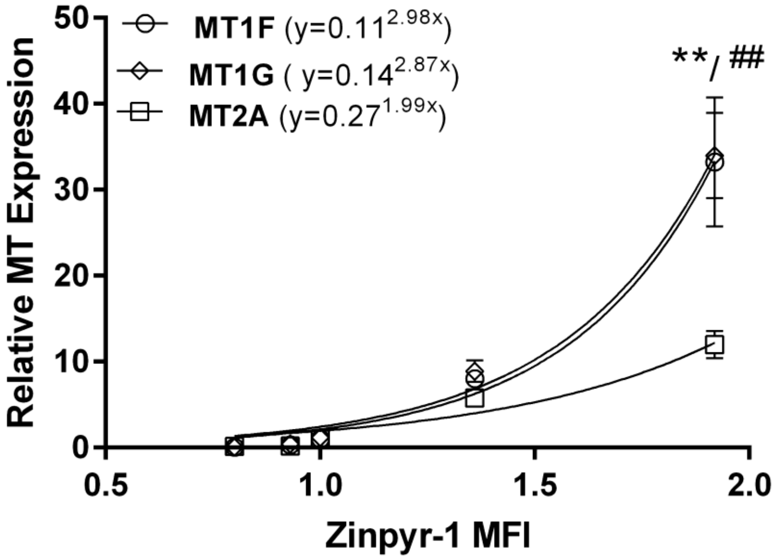

Supplementary Figure 2. Zinc mediated metallothionein induction rates.

MT response to intracellular zinc was calculated by plotting MT expression for MT1F, MT1G and MT2A against zinpyr-1 median fluorescent intensity (MFI). Exponential induction of the MT1 genes was far superior to MT2A as represented by their exponential growth rate. Data are representative of two independent experiments. * MT1F versus MT2A, # MT1G versus MT2A expression. Mann-Whitney test, **/## $p<0.01$ (mean ± SE).

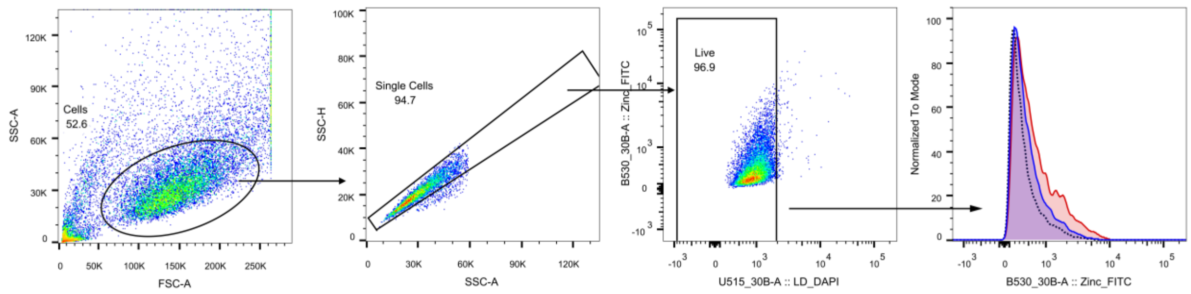

Supplementary Figure 3. Gating strategy for intracellular zinc quantification.

To quantify intracellular zinc, zinpyr stained cells were examined by flow cytometry according to the following gating strategy. Single cells were gated using forward and side scatter, followed by live cell selection represented by cells negatively stained by the viability stain within the DAPI channel. FITC median fluorescence intensity was quantified in live Huh-7 (Figure 2B) or Caco-2 cells (Figure 7A).

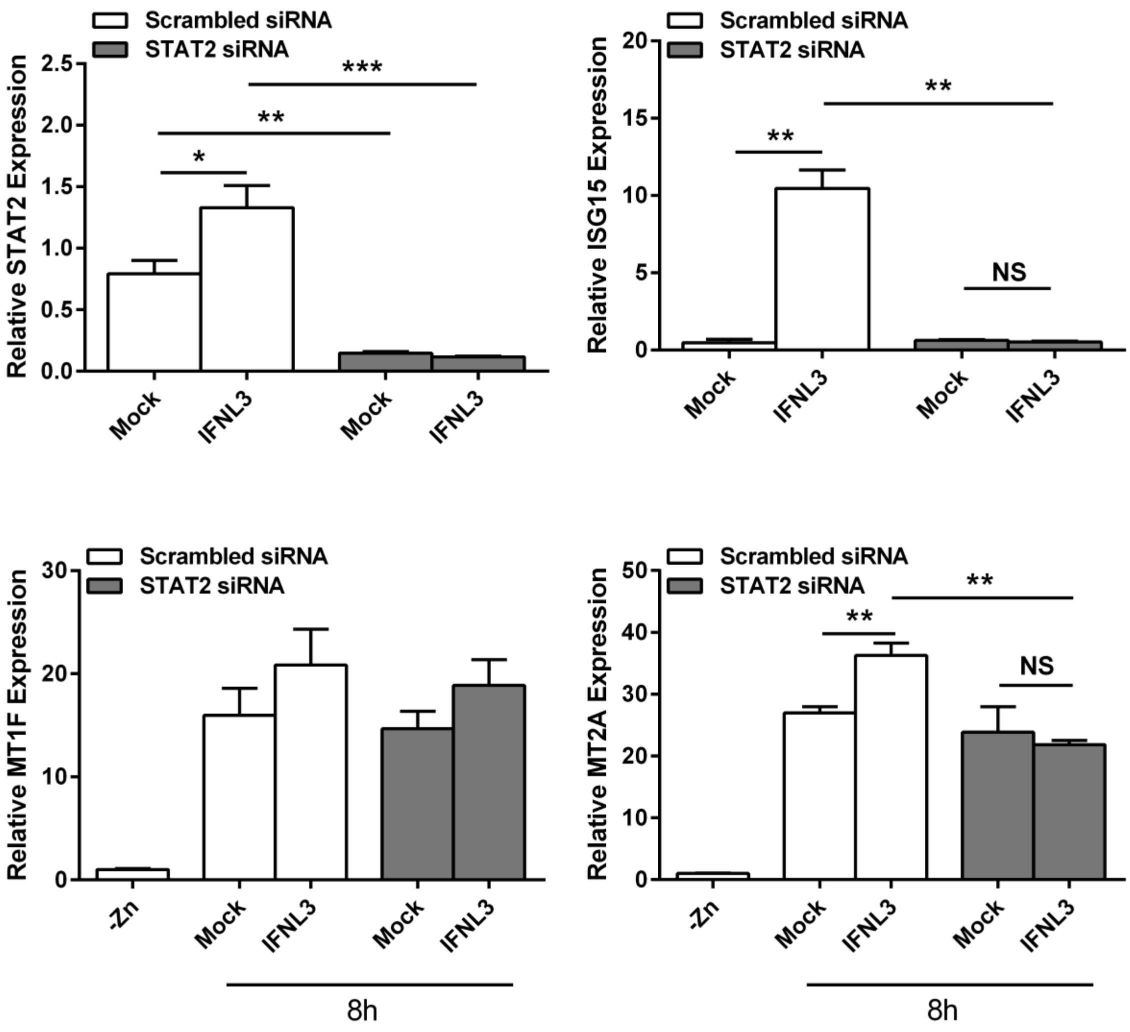

Supplementary Figure 4. STAT2 knockdown does not affect MT1 gene expression

To examine the role of classical STAT1/2 mediated IFN signalling, Huh-7 cells were treated with 10nM siRNA targeting STAT2 and a scrambled siRNA as a control, then treated with 100ng/ml IFNL3 for 8 h with 5 µM $ZnSO_4$. STAT2 knockdown (>80%) resulted in the disruption of IFNL3 mediated ISG15 and MT2A expression with no effect on IFNL3 mediated MT1F expression. An additional control lacking zinc was included with MT1F/MT2A expression data to demonstrate MT induction following zinc treatment. Data are representative of two independent experiments. * p<0.05, ** p<0.01, *** p<0.001, NS not significant, (mean ± SE).

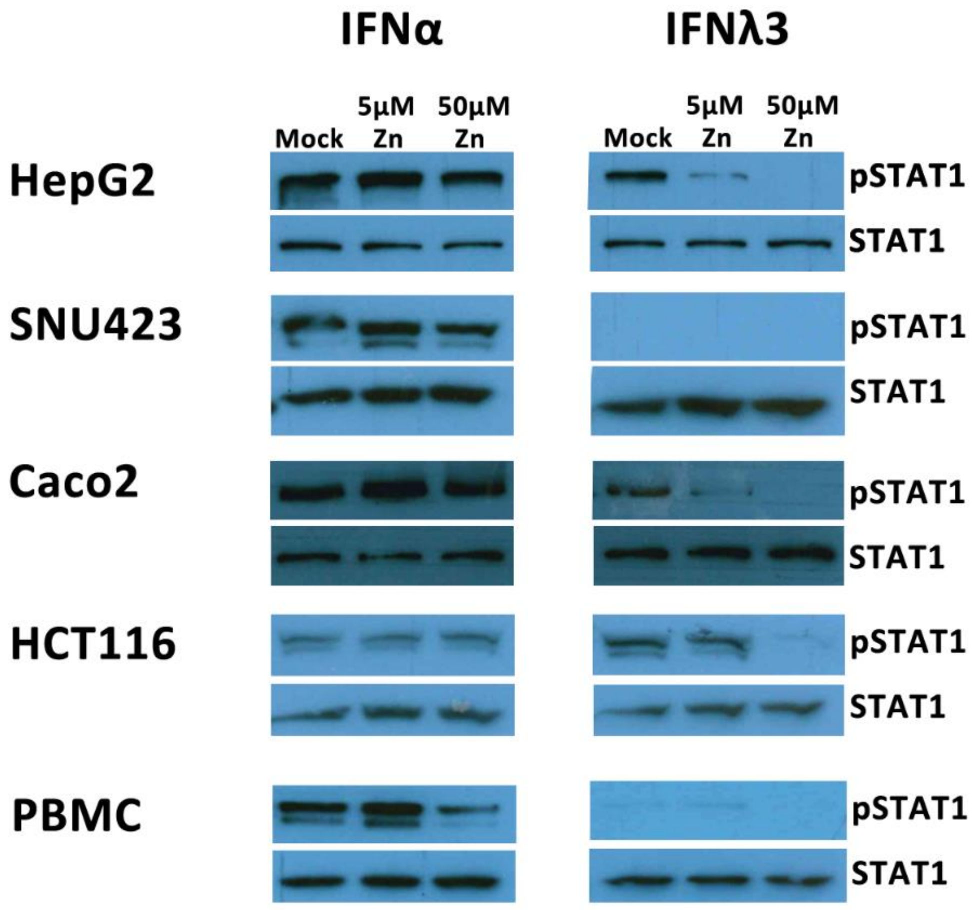

Supplementary Figure 5. Zinc mediated inhibition of IFNL3 response in different cell lines

Hepatoblastoma HepG2, hepatocellular carcinoma SNU423, colorectal carcinoma cell lines Caco2 and HCT116 and fresh PBMCs were treated with IFNA or IFNL3 in the presence of 0, 5 and 50 µM $ZnSO_4$. Unlike IFNA, zinc dose-dependently inhibited IFNL3 STAT1 phosphorylation at 30 minutes post-treatment. 5 µM $ZnSO_4$ potentiated IFNA mediated STAT1 phosphorylation in most cell lines and was only inhibitory in PBMCs at 50 µM concentration. All cells examined were responsive to IFNL3 except SNU423 cells which demonstrated no STAT1 phosphorylation post-IFNL3 treatment. Data are representative of one experiment.

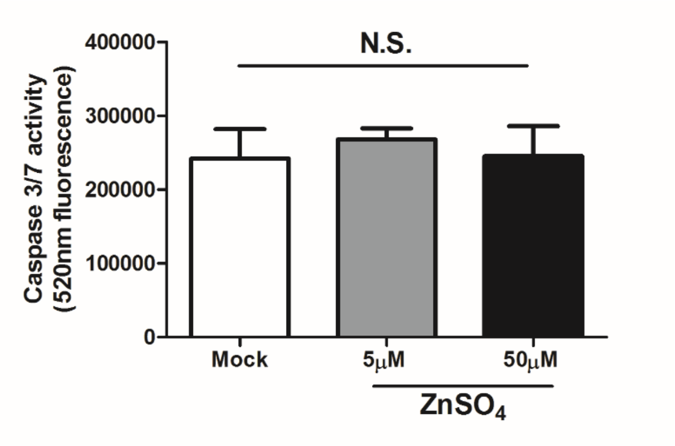

Supplementary Figure 6. Zinc treatment does not affect cell viability

To ensure that zinc does not promote Huh-7 cell death, cells were treated for 24 h with 5μM and 50μM $ZnSO_4$ and caspase 3/7 activation was measured according to the manufacturers protocol (FAM FLICA Caspase 3/7 Assay Kit, Immunochemistry technologies). No increase in caspase activity and thus apoptosis was detected at either concentration of zinc. Data are representative of two independent experiments (Welch's t test). NS not significant, (mean ± SE).

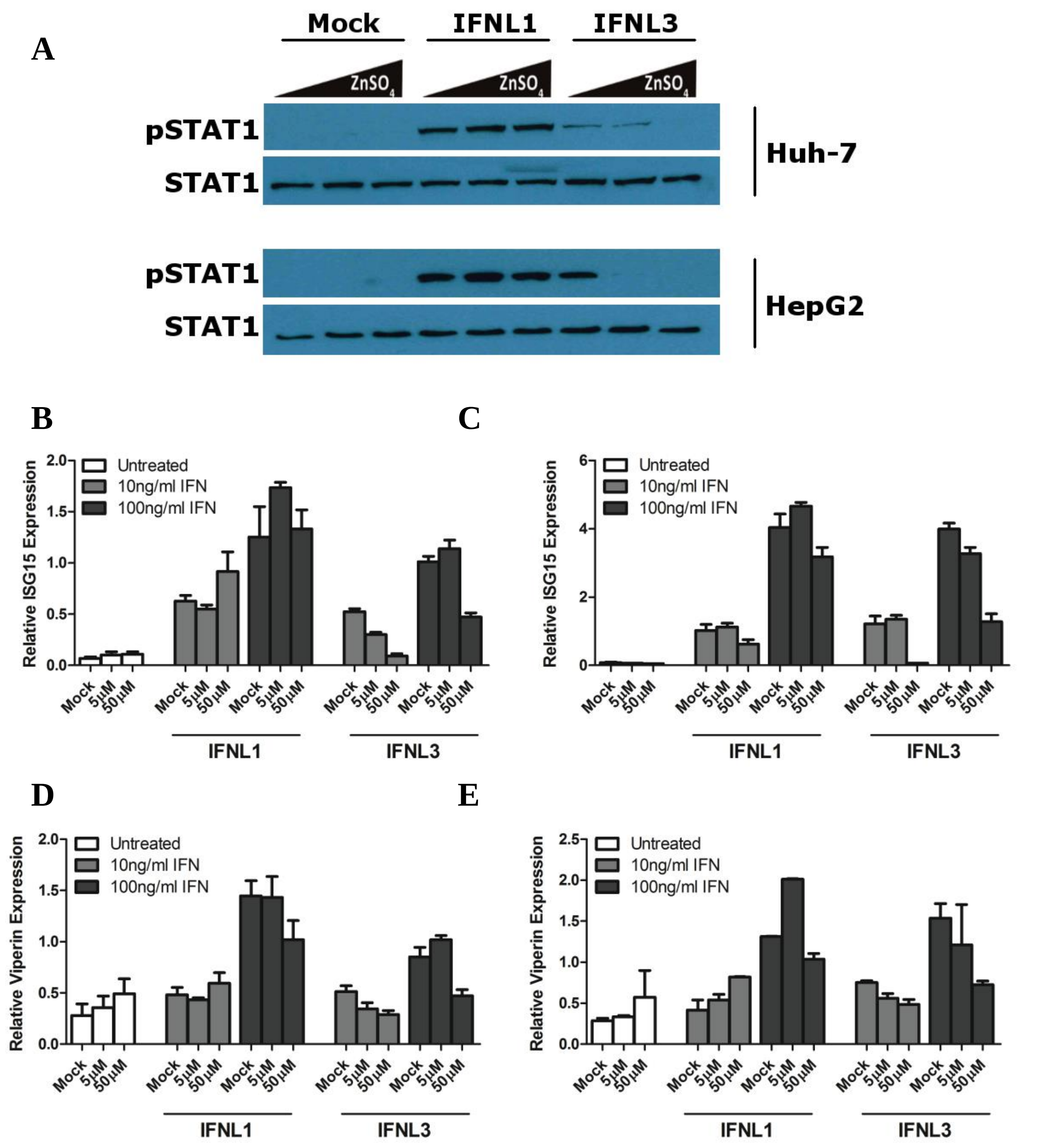

Supplemental Figure 7. Zinc inhibits IFNL3 but not IFNL1

Huh-7 and HepG2 cells were pre-treated with 0, 5 and 50μM $ZnSO_4$ for 24 h, then treated with 100ng/ml IFNL1 or IFNL3 for 15 min, and STAT1 phosphorylation was measured (A). IFNL3 mediated STAT1 phosphorylation was dose-dependently decreased by zinc, whereas zinc had no effect on STAT phosphorylation mediated by IFNL1. A similar inhibition of IFNL3 but not IFNL1 mediated ISG15 and viperin expression was observed at 8 h (B, D) and 24 h (C, E) (1 experiment). Neither concentration of $ZnSO_4$ reduced IFNL1 mediated ISG expression, however both 5 μM and 50 μM $ZnSO_4$ dose-dependently inhibited IFNL3 mediated ISG15 and viperin. Data are representative of one experiment performed in duplicate (mean ± SE). No statistical analysis performed.

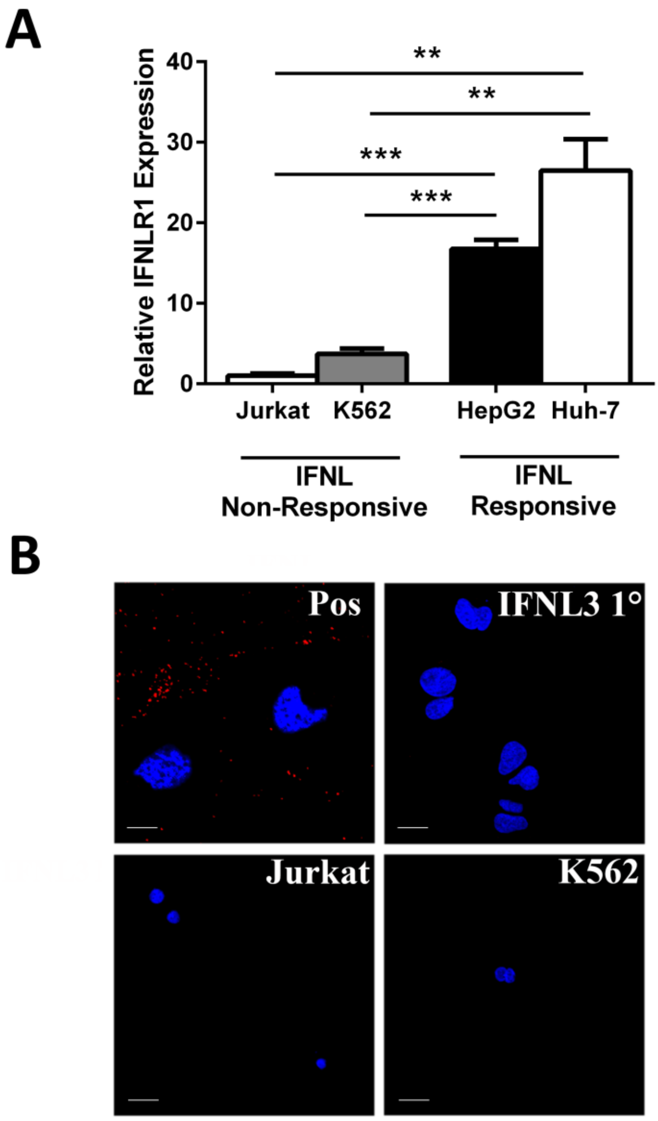

Supplementary Figure 8. Proximity ligation assay (PLA) negative controls

To ensure the validity of PLA assays, multiple negative controls were performed with the IFNL3 primary antibody alone, and with cell lines that are not responsive to IFNLs. Compared to IFNL responsive cell lines HepG2 and Huh-7, non-responsive cell lines Jurkat and K562 [11] express significantly lower levels of IFNLR1, as measured by qPCR (A). Due to an absence of surface IFNLR1 expression, Jurkat and K562 cells produced no PLA signal, (B). Additional negative control PLAs were performed with all the PLA reagents using the primary IFNL3 antibody without the IFNLR1 antibody. Scale bars represent 10 μm. Data are representative of two independent experiments. * $p<0.05$, ** $p<0.01$, *** $p<0.001$, (mean ± SE).

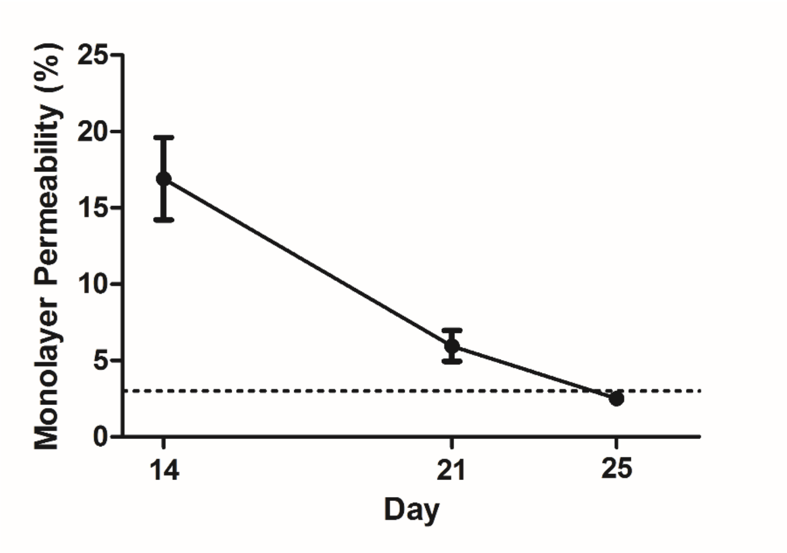

Supplementary Figure 9. Measurement of Caco-2 monolayer permeability with fluorescein

Differentiated Caco-2 monolayer integrity was measured at day 14, 21 and 25 using the cell impermeable dye fluorescein. Monolayer permeability was calculated as bottom well fluorescence/upper transwell fluorescence x 100, with a value below 3% considered adequate monolayer integrity. Values below 3% were achieved at day 25, and subsequently zinc transport experiments were performed. Data are representative of two independent experiments (mean ± SE). No statistical analysis performed.

MT2A

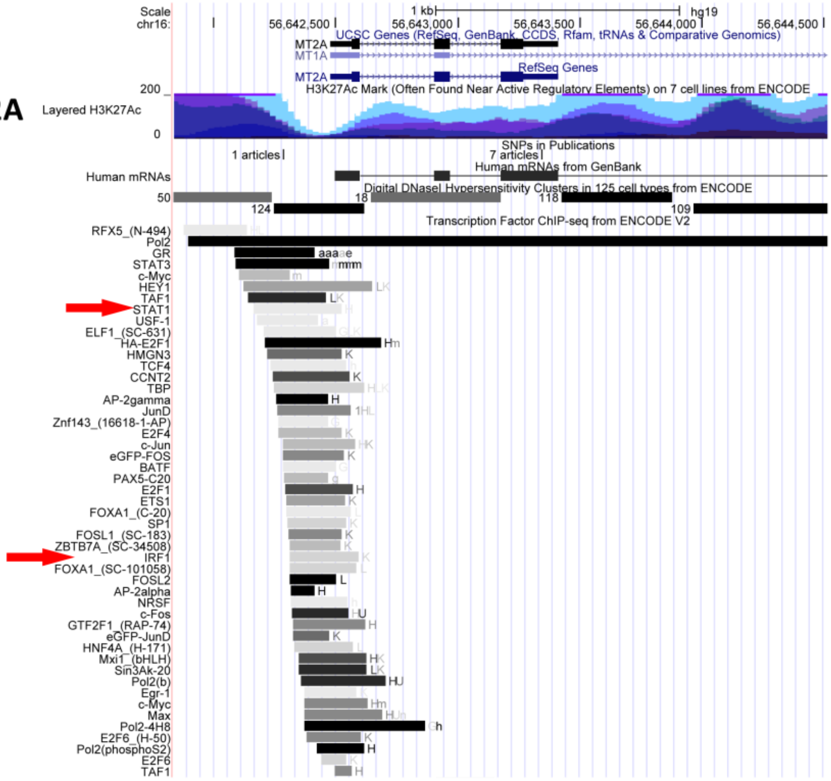

MT1F

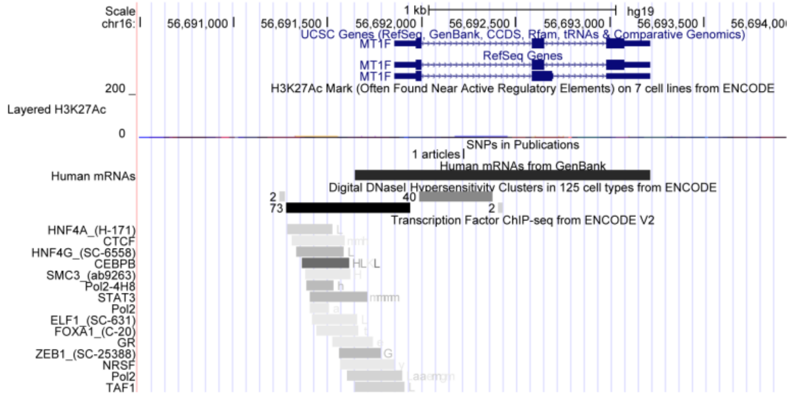

MT1G

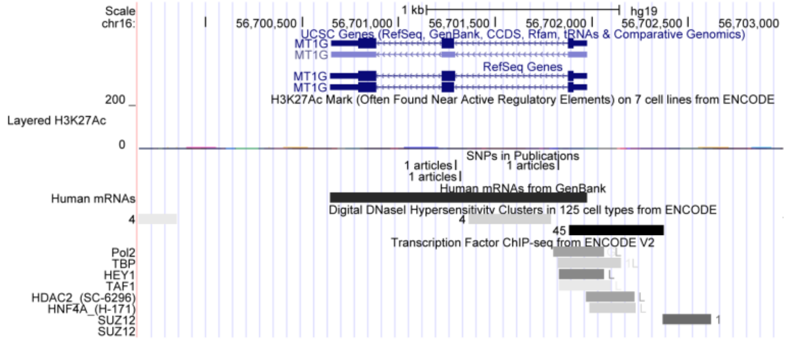

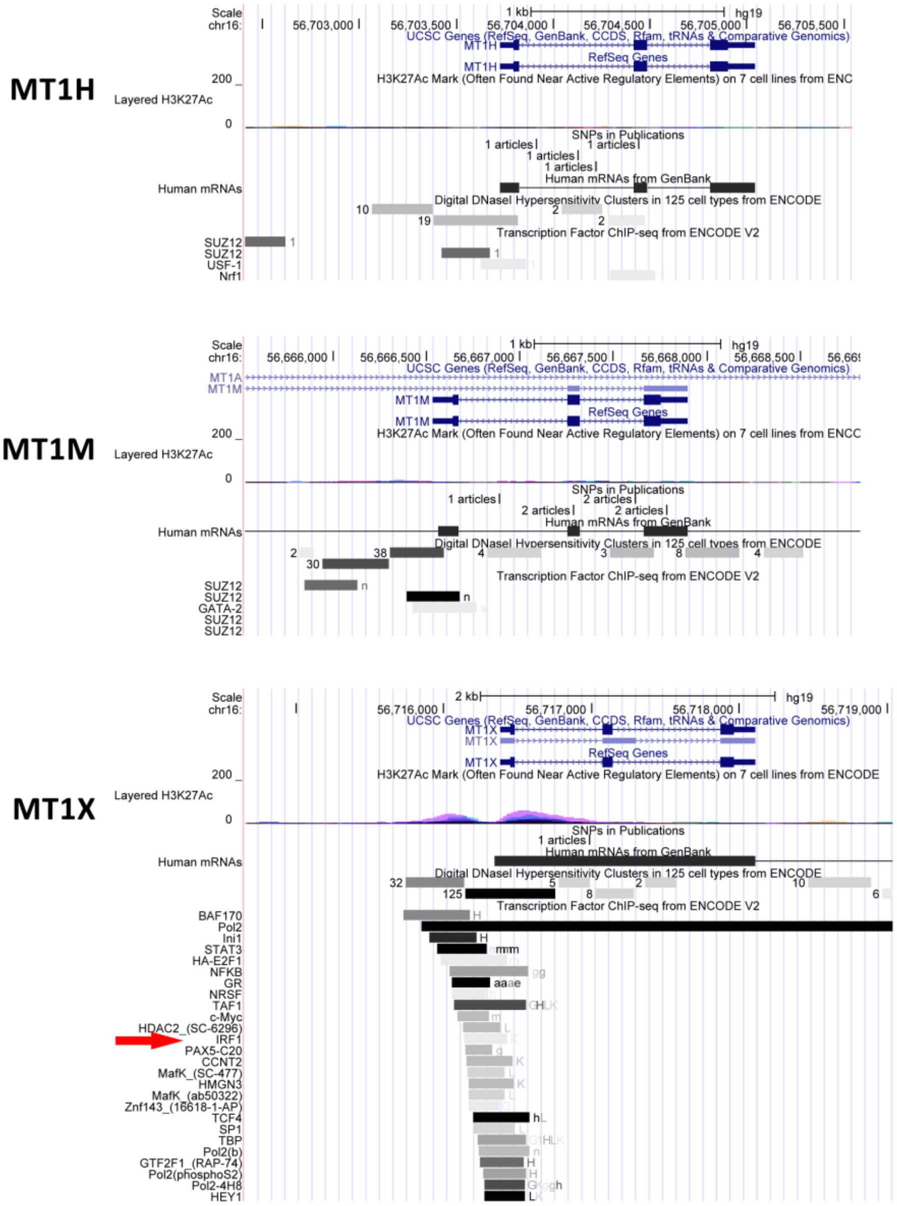

Supplementary Figure 10. Validated transcription factor binding sites in MT promoter regions.

Transcription factor binding sites within MT promoters and enhancers were examined using the UCSC genome browser [12]. Compared to MT2A, MT1 family members demonstrate a large reduction in transcription factors that are activated by inflammation and immunity: STAT1, IRF1, STAT3, NFκB and AP-1. Signalling components activated by type I/III IFN, STAT1 and IRF1 (red arrows), were present only in MT2A and MT1X, suggesting direct activation by IFNs.

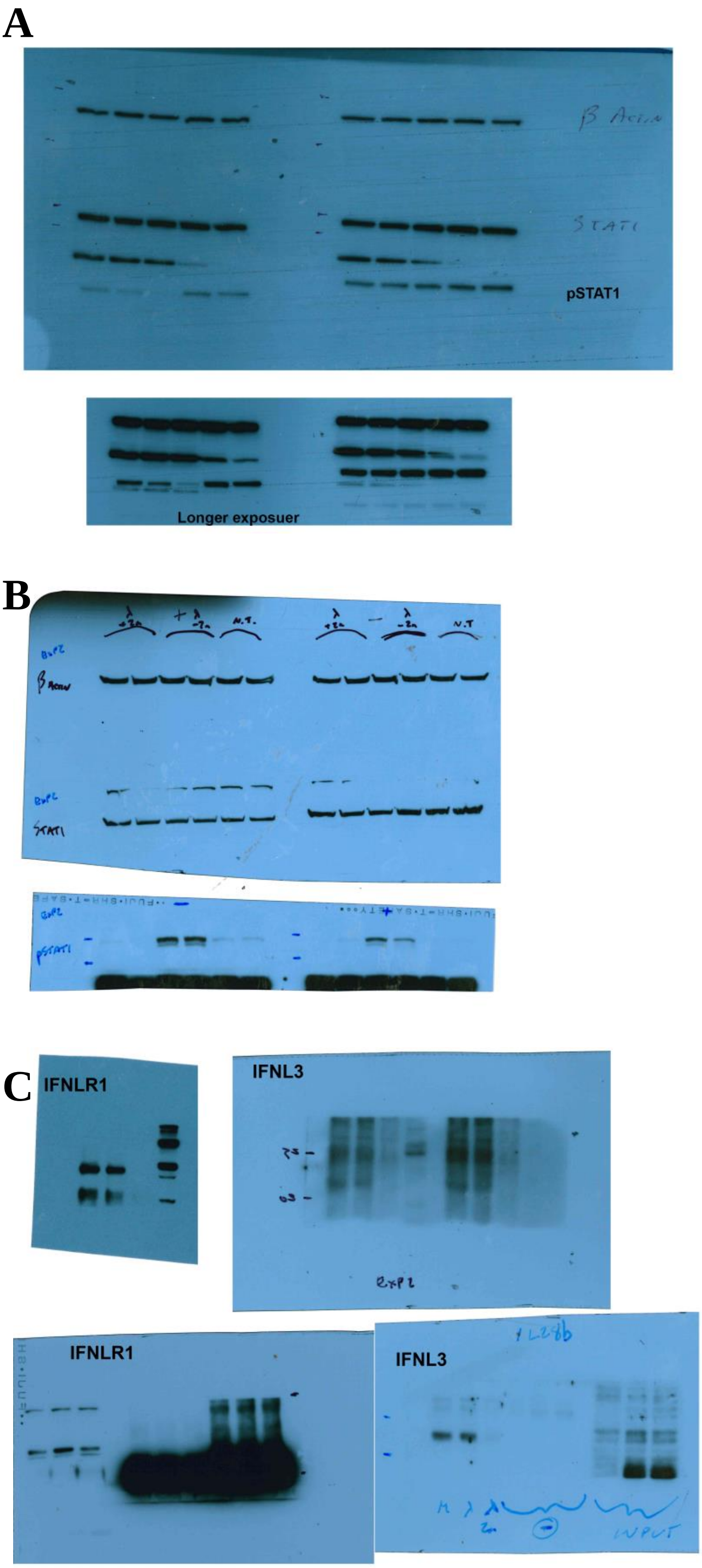

Supplementary Figure 11. Full Western Blots

Full Western blots for Figures 3A (A), 6A (B) and 6C (C).

Supplementary Table 1. ISGs from Figure 4C that are listed as within the top 25 antiviral ISGs for each virus (Schoggins et al.)

**Huh7**	**Huh7.5**	**STAT1-/- Fibroblast**	**STAT1-/- Fibroblast**	**STAT1-/- Fibroblast**	**Huh7**	**T Cells**
**HCV-48h**	**HCV-48h**	**WNV**	**VEEV**	**CHIKV**	**YFV**	**HIV**
MAP3K14	SIRPA	EHD4	UNC93B1	MAP3K14	HES4	MCOLN2
HES4	GZMB	UNC93B1	SIRPA	EHD4	CCL2	CD74
GBP2	CCL2	IFI16	LGALS9	UNC93B1	SLC25A28	
BLVRA	CCDC109B	RIGI	SCARB2	SLC25A28	NT5C3	
NOD2	MAB21L2		RIGI	RIGI	LAP3	
PARP12	TNFAIP6					
NT5C3	IFIT5					
RIGI	RIGI					

Supplementary Table 2. Disulphide analysis of alkylated cysteines in the IFNL3 protein.

IFNL3 Treatment	IFNL3 Disulphide Bond	Ion Score	Peptide	Modification	Disulphide Status
IAA	Cys37-Cys136	40	R.G**C**HIAQFK.S	x1 CAM	Reduced
IAA	Cys71-Cys169	37	K.KESPG**C**LEAVSVTFNLFR.L	x1 CAM	Reduced
IAA	Cys188-Cys195	36	R.DLN**C**VASGDL**C**VHHHHHH.-	x2 Dehydro	Oxidised
Zn + IAA	Cys37-Cys136	22	G.G**C**HIAQFK.S	x1 CAM	Reduced
Zn + IAA	Cys71-Cys169	50	K.KESPG**C**LEAVSVTFNLFR.L	x1 CAM	Reduced
Zn + IAA	Cys188-Cys195	23	R.DLN**C**VASGDL**C**VHHHHHH.-	x2 Dehydro	Oxidised
DTT + IAA	Cys37-Cys136	34	R.G**C**HIAQFK.S	x1 CAM	Reduced
DTT + IAA	Cys71-Cys169	155	K.KESPG**C**LEAVSVTFNLFR.L	x1 CAM	Reduced
DTT + IAA	Cys188-Cys195	106	R.DLN**C**VASGDL**C**VHHHHHH.-	x2 CAM	Reduced

Supplementary Table 3. Redox state of IFNL3 disuplide bonds

IFNL3 disulphide bond	Native state	Percent oxidised + 10mM ZnSO4	Percent oxidised + 10 mM EDTA
Cys37-Cys136	100	99.7	99.7
Cys71-Cys169	93.1	92.6	90.3
Cys188-Cys195	98.5	98.4	97.3

Supplementary References

1 Jakob, U., Muse, W., Eser, M. & Bardwell, J. C. Chaperone activity with a redox switch. *Cell* **96**, 341-352, (1999).

2 Briggs, D. B. *et al.* Zinc enhances adiponectin oligomerization to octadecamers but decreases the rate of disulfide bond formation. *Biometals* **25**, 469-486, (2012).

3 Erlandsson, M. & Hallbrink, M. Metallic zinc reduction of disulfide bonds between cysteine residues in peptides and proteins. *Int J Pept Res Ther* **11**, 261-265, (2005).

4 Bekendam, R. H. *et al.* A substrate-driven allosteric switch that enhances PDI catalytic activity. *Nature communications* **7**, (2016).

5 Gad, H. H. *et al.* Interferon-lambda is functionally an interferon but structurally related to the interleukin-10 family. *J. Biol. Chem.* **284**, 20869-20875, (2009).

6 Babor, M., Gerzon, S., Raveh, B., Sobolev, V. & Edelman, M. Prediction of transition metal-binding sites from apo protein structures. *Proteins-Structure Function and Bioinformatics* **70**, 208-217, (2008).

7 Buchan, D. W. A., Minneci, F., Nugent, T. C. O., Bryson, K. & Jones, D. T. Scalable web services for the PSIPRED Protein Analysis Workbench. *Nucleic Acids Res.* **41**, W349-W357, (2013).

8 Zhao, W. *et al.* Structure-based de novo prediction of zinc-binding sites in proteins of unknown function. *Bioinformatics* **27**, 1262-1268, (2011).

9 Ebert, J. C. & Altman, R. B. Robust recognition of zinc binding sites in proteins. *Protein Sci.* **17**, 54-65, (2008).

10 Sheahan, T. *et al.* Interferon lambda alleles predict innate antiviral immune responses and hepatitis C virus permissiveness. *Cell Host Microbe* **15**, 190-202, (2014).

11 Ding, S., Khoury-Hanold, W., Iwasaki, A. & Robek, M. D. Epigenetic reprogramming of the type III interferon response potentiates antiviral activity and suppresses tumor growth. *PLoS biology* **12**, e1001758, (2014).

12 Kent, W. J. *et al.* The human genome browser at UCSC. *Genome Res.* **12**, 996-1006, (2002).
